# Supplementary material for: The Role of Akkermansia muciniphila on Improving Gut and Metabolic Health Modulation: A Meta-Analysis of Preclinical Mouse Model Studies
Source: Microorganisms. 2024 Aug 9;12(8):1627. doi: 10.3390/microorganisms12081627 (PMC11356609; doi:10.3390/microorganisms12081627)
Supplement: Supplementary file 1 [file microorganisms-12-01627-s001.zip › microorganisms-3150450-supplementary.pdf]

Supplementary Data

Table S1. Characteristics of the studies

N	Author, year, city, country	Disorder/ condition	Strain	Sex	Age	*AKK* strain	Diet	Intervention	Overall conclusion
**Age-related conditions**									
1	Cerro 2022. Madrid, Spain	Healthy aging; lifespan	ICR-CD1	F	40 & 72 w	CIP107961T; live	A04	2×10^8 cfu daily for 4 w; gavage	*Akk* improved immune function, antioxidant capacity, inflammation, and increased lifespan.
2	Ma 2023, Shanghai, China	Age-related disorders	C57BL/6	M	72 w	BAA-835; live	Chow	FMT *Akk* 3×10^9 cfu daily for 4 w and every other day from 2 to 12 w; gavage	*Akk* is reduced in old mice; FMT with *AKK* in old mice improved glucose sensitivity, hepatospleno-megaly, inflammaging, antioxidative capacity and intestinal barrier and increased acetate.
3	Van Der Lugt 2019, Wageningen, The Netherlands	Age-related decline in intestinal health and lifespan	$Ercc1^{-/\Delta7}$	M & F	16 w	BAA-835; live	D12450B purified (Research Diets)	2×10^8 cfu 3 times per w for 10 w; gavage	*Akk* increased thickness of colonic mucus layer; reduced inflammation in colon and ilium; no effect on survival or BW.
**GI disorders**									
4	Wang 2022, Tianjin, China	DSS-induced colitis<br>Intestinal radiation toxicity	C57BL/6J	M	7 w	BAA-835; live<br>heat inactivated<br>modified *Akk*@MFe_3O_4	NR	5×10^7, 2.5×10^8 cfu for 3 d after DSS or radiation; oral	Only the live bacteria show therapeutic effects. *AKK*@MFe_3O_4 was more effective. Improved BW, DAI[1] score, colon length; reduced inflammation; increased IL10 and *Lactobacillus murino.*
5	Kang 2013, Seol, South Korea	DSS-induced colitis	C57BL/6	F	NR	BAA-835; live<br>EVs[2]	NR	5 x 10^8 cfu, live; 100 µg EVs together with DSS for 8 d.	Both interventions improved body weight and colon length; reduced inflammation and DAI score.
6	Ring 2019, Nuthetal, Germany	Intestinal inflammation induced by IL10 deficiency	C57BL/6.129P 2-$Il10^{tm1Cgn}$ Germ free	M	8 w	BAA-835; live	Irradiated Chow	Germ-free + *Akk* $1\text{x}10^8$ cfu once, gastric gavage<br>Germ-free + *Akk* + *E. coli* NC101<br>SIHUMI + *Akk*	*Akk* do not induce pro-inflammatory effects in IL10-deficient mice colonized with *E. coli* or a humanized microbiome (SIHUMI). Increased colon length and BW.
7	Yu 2022, Beijing, China	Restrain stress<br>High fructose (20%)	C57BL/6J	F	8 w	BAA-835; live	NR	1×10^8 cfu, daily for 2 w together with intervention; gavage	*Akk* improved intestinal barrier; increased NLRP6[3] function, autophagy, and expression of TJ proteins; reduced NF-kB activity and inflammation.
8	Qian 2022, Anhui, China	DSS-induced colitis	C57BL/6	M	5-6 w	Amuc_2109; protein	NR	20 µg, daily for 3 w; oral gavage	Amuc_2109 improved DAI scores and colon length; reduced weight loss, oxidative stress, inflammatory cytokines; restored the mRNA expression of TJ proteins and reshaped the intestinal microbiota.

[1] disease activity index

[2] extracellular vesicles

[3] nucleotide-binding oligomerization domain–like receptor 6

N	Author, year, city, country	Disorder/ condition	Strain	Sex	Age	*AKK* strain	Diet	Intervention	Overall conclusion
9	Liu 2021, Wuxi, China	DSS-induced colitis	C57BL/6 and SPF[4]	M	adult	BAA-835; live<br>FSDLZ20M4; live<br>FSDLZ36M5; live<br>FSDLZ39M14; live	Standard commercial mouse food	1×10^9 cfu, daily for 1 w, oral	Only FSDLZ36M5 restored colon length, BW, reduced gut permeability, and increased anti-inflammatory cytokine, and remodeled the gut microbiota back to control.
10	Bachmann 2022, Brussels, Belgium	Survival and wound healing in colon perforation	C57BL/6J WT or IEC-MyD88KO[5] and SOPF[6]	M	9 w	BAA-835; live	NR	1×10^9 cfu, daily for 3 w, oral gavage	*Akk* improved wound healing, survival and IL22 expression through a mechanism requiring the MyD88-dependent pathway. However, it did not affect body weight, mucus thickness, and Muc2 expression.
		Human trial (3 control and 7 intervention)		NR	NR			1×10^{11} cells/day colon catheter (continuous 2 h)	No side effects after 1 w; *Akk* increased expression of TRIM40[7], and MARCO[8].
11	Zhai 2019, Shanghai, China	DSS-induced colitis	C57/BL6 and SPF	M	8 w	ATCC-BAA-835; live<br>139 (murine strain); live	NR	2×10^8 cfu, daily for 46 d, gavage	ATCC strain had a greater anti-inflammatory capacity improving spleen weight, colon inflammation index, and histological score; reduced TNF-α and IFN-γ in colon; normalization of the gut microbiota; increased differentiation of Tregs and SCFAs production mainly acetate.
12	Kim 2021, Seoul, South Korea	Stem cell-mediated epithelial development	SPF C57BL/6 and Lgr5-EGFP-IRES-CreERT2 (Lgr5-GFP) reporter gene	F	8-10 w	BAA-835; live<br>BAA-835; pasteurized<br>AK32 (KCTC 14172BP from human feces	Sterile food and water	8×10^8 cfu daily for 4 w; oral gavage in control, radiation, and methotrexate-exposed mice	*Akk* increased Muc2 and mucus thickness and differentiation of Paneth and goblet cells; elevated cecal acetate and propionate, and reduced gut damage induced by radiation and methotrexate.<br>There was no effect with pasteurized *Akk*. Superior effects with AK32 vs. BAA-835.
13	Chen 2022, Anhui, China	Intestinal mucositis induced by 5-FU[9]	C57BL/6 and SPF	M	5-6 w	BAA-835; live	NR	1.5×10^9 cfu daily for 4 w, gavage	Live and Amuc_100 protein restored colon length, improved intestinal barrier, reduced colon inflammation (TNFα, IL6, NLRP), the F/B ratio and increased IL10.
						Amuc_1100, protein		100 μg daily for 4 w, gavage	
14	Xie 2022, Nanjing, China	Cadmium-induced intestinal mucosal damage	C57BL/6	M	4 w	Live *Akk*<br>Pasteurized *Akk*	NR	1×10^7 cfu; once a day for fifteen days; oral	*Akk* improved bodyweight and colon length, and declined inflammation, improved melatonin production through increase in enterochromaffin cells number. Moreover, it caused prevention of the toxic effects by increasing the goblet cells and scavenging the ROS.
15	Qu 2021, Hangzhou, China	DSS-induced colitis	SPF C57BL/6 WT and $NLRP3^{tm1Bhk}$ ($NLRP3^{-/-}$)	M	6-8 w	BAA-835; live	NR	1×10^9 cfu daily for 7d; oral gavage in antibiotic treated mice	*Akk* improved body weight, colon length; increased gobbler cells, mucin, and IL1β; and reduced inflammation.

[4] specific pathogen free

[5] intestinal epithelial specific MyD88 knock out.

[6] specific and opportunistic pathogen-free

[7] tripartite motif containing 40.

[8] macrophage receptor with collagenous structure

[9] 5-fluorouracil

N	Author, year, city, country	Disorder/ condition	Strain	Sex	Age	*AKK* strain	Diet	Intervention	Overall conclusion
16	Bian 2019, Hangzhou, China	DSS-induced colitis	SPF C57BL/6	M	6-7 w	BAA-835; live	NR	$3×10^9$ cfu daily for 14 d; oral gavage	*Akk* improved body weight, colon length, gut barrier function; reduced inflammation; increased acetate, propionate, butyrate, and isobutyrate; and increased *Verrucomicrobia, Akkermansia, Ruminococcaceae,* and *Rikenellaceae* abundance
17	Gu 2021, Nanjing, China	DSS-induced colitis	C57BL/6J	M	6-8 w	BAA-835; live<br>BAA-835 pasteurized<br>Amuc_1100, protein	NR	$1.5×10^8$ cfu for 10 d, gavage live and pasteurized<br>3 µg daily for 10 d, gavage	Patients with UC and mice with colitis have reduced Trp, Kyn, 5-HT and 5-HTP and reduced *Akk*. *Akk* regulated Tryptophan metabolism by blocking the Kynurenine and activating the microbial Indole pathway. All *Akk* interventions increase IL10, IL22, and CYP1A2
**Metabolic disorders & HFD-feeding**									
18	Li., 2016, Hong Kong, China	HFD-induced atherosclerosis	$ApoE^{-/-}$	M	8 w	BAA-835, live<br>BAA-835, heat killed	Chow or Western diet, D12079BResearch Diets	$5 × 10^9$ cfu, daily for 8 w; gavage	Only live *Akk* reduced plaque. No effect on lipid profile or glucose tolerance. *Akk* reduced macrophages, MCP1, TNFα and ICAM1 in plaque; and serum MCP1, IL1β and sTNFR II. *Akk* increased occluding, and ZO1 and reduced serum LPS. Infusion with LPS prevented *Akk* effects.
19	Grander 2018, Innsbruck, Austria	Alcoholic liver disease	C57BL/6 and Lieber-DeCarli diet (1-5 vol% ethanol)	F	7-8 w	Muc^T CCUG 64013, live	0-5% EOH for 15 d	$1.5×10^9$ cfu twice before EtOH or every other day with EtOH or days 10, 12 and 14 of 0-5% EtOH for 15 d	*Akk* reduced liver ALT, IL1β, MPO^+ cells, TG, Liver weight and serum LPS; increased mucus thickness, goblet cell number and claudin and Occludin expression.
20	Wu 2019, Guangzhou, China	HFD-induced metabolic disorders	C57BL/6	F	8 w	*Akk* ATCC BAA-835	HFD	10^9 cfu, 10 months, gavage	*Akk* reduced body weight gain, blood glucose, and insulin resistance and increased IL10 level. Moreover, *Akk* increased the relative fecal abundance of *Bifidobacterium* and was negatively correlated with the fecal abundance of Bacteroides.
21	Nian 2023, Shanghai, China	HFD-induced NAFLD	SPF C57BL/6	M	NR	*Akk,* NR<br>*Bifidobacterium bifidum,* NR	HFD, 40% fat for 20 w	$1 × 10^9$ cfu; 3 times/w for 8 w into antibiotic-treated mice; gavage	*Akk* and BB alone or together showed same effects: attenuation of body weight gain, insulin resistance, liver steatosis; serum ALT, AST TG, TC, liver TG and TC; serum inflammation; and tight-junction protein expression. FXR was upregulated in the liver and reduced in the intestine. The interventions improved α and β diversity in gut.
22	Plovier 2017, Brussels, Belgium	Obese and diabetic mice	C57BL/6J	M	10-11 w	*Akk* grown on mucus-based medium<br>*Akk* grown on synthetic medium<br>Pasteurized *Akk*<br>Live *Akk* grown on synthetic medium<br>Pasteurized *Akk* grown on synthetic medium<br>Protein Amuc_1100	HFD	$2×10^8$ cfu; 4 w; oral gavage	*Akk* maintains its effectiveness when cultivated using a synthetic substrate suitable for human intake. Subjecting *Akk* to pasteurization enhanced its ability to reduce fat mass, BW gain, food intake, plasma glucose, plasma insulin, insulin resistance, and abnormal lipid levels. Amuc_1100 enhanced intestinal integrity and partially mimics the advantageous outcomes of the microorganism. Both live and pasteurized forms of *Akk* cultivated on the synthetic medium, are deemed safe for human use.

N	Author, year, city, country	Disorder/ condition	Strain	Sex	Age	*AKK* strain	Diet	Intervention	Overall conclusion
23	Yoon 2021, Seoul, Korea	HFD-fed mice	C57BL/6J	M	6 w	Purified protein from *Akk*, P9	HFD	100 μ; 8 w; oral administration	The intervention increased thermogenesis through induction of UCP1 in brown adipose tissue and systemic GLP-1 expression; improved weight gain, food intake, oral glucose tolerance, and glucose homeostasis.
24	Deng 2020, Guangdong, China	HFD-murine model	C57BL/6	M	NR	*Akk* I (Amuc_GP01)<br>*Akk* II (Amuc_GP25)	HFD	5×10^9 cfu; 16 w; oral gavage	Amuc_GP01 improved impaired glucose tolerance, hyperlipidemia and liver steatosis through inhibiting brown adipose tissue whitening and inflammation induced by HFD, by repairing the intestinal barrier and relieving endotoxemia. Amuc_GP25 was not as effective as Amuc_GP01.
25	Morrison 2022, Leiden, The Netherlands	HFD-induced NASH[10]	$LDLR^{-/-}$ Leiden C57BL/6	M	15-17 w	BAA-835, pasteurized	HFD, 45% fat	2×10^8 cfu/mouse in diet for 28 w	No effect on NASH, liver fibrosis, BW, lipids, glucose, insulin, or bile acids. *Akk* reduced MIP1α in ilium and KC in colon; improved gut barrier function; reduced valeric and caproic acid, reduced *Prevotella* and increased *Parasutarella* and *Bifidobacterium* in ilium and in the colon; reduced *Corynebacterium*, *Desulfovibrionales* and *Dorea* and increased *Enterorhabdus* and *Bifidobacterium.*
26	Yang 2020, Wanju-gun, Korea	Obesity	C57BL/6	M	7 w	*Akk* type strain BAA 835<br>*Akk* 10<br>*Akk* 19<br>*Akk* 27	HFD	1×10^8 cfu; oral gavage; 12 w (6 times per week)	The three strains improved metabolic imbalances caused by HFD, addressing factors such as BW gain, caloric intake, adipose tissues weight, total fat mass, glucose homeostasis, and insulin sensitivity. These outcomes were associated with reduced low-grade intestinal inflammation, restoration of a healthy gut microbiome and gut integrity and prevention of liver steatosis.
27	Katiraei 2020, Leiden, The Netherlands	HCD-induced hyperlipidemia and Cuff-induced neointima formation	ApoE*3-Leiden	M	9-13 w	BAA-835, live	Western diet, 1% Cho and 0.05% cholate for 7 w	2×10^8 cfu; live gavage daily for 4 w after week 3 of HC diet	*Akk* reduced BW, plasma TC, and TG; increased B cell and reduced T cell in mesenteric lymph nodes
28	Kim 2020, Seoul, South Korea	HFD-induced NAFLD and fibrosis	C57BL/6N	M	5 w	BAA-835, live	HFD, 45% fat, LFD diet 10% fat	1×10^8-1×10^9; gavage daily for 10 w	*Akk* reduced serum TG, ALT, and liver SREBP and IL6, liver damage and fat deposition; improved F/B ratio and microbiome diversity. No effect on BW.
29	Keshavarz Azizi Raftar 2021, Tehran, Iran	HFD/CCl4-induced Liver Injury	C57BL/6	M	7-8 w	BAA-835, live<br>BAA-835, pasteurized<br>BAA-835, EVs	HFD, 60% fat and 10% CCL4 for 4 w before AKK	1×10^9 cfu; 4 w; gavage<br>1×10^9 cfu; 4 w; gavage<br>50 μg; 4 w; gavage	All interventions reduced liver weight and fibrosis; serum TNFα, IL6, ALT, AST, glucose, TG, TC, VLDL and LDL; reduced inflammation in liver and colon; increased ZO1 in colon and serum IL10 and HDL; increased *Bifidobacteriumspp, Roseburia, Methanobre-vibacter, Alistipes, Veillonella,* and *Faecalibacte-rium prausnizii*.
30	Zhao 2017, Shanghai, China	Chow diet-fed mice	C57BL/6	M	6 w	*Akk* (ATCC BAA-835)	normal chow diet	2×10^8 cfu; 5 w; oral gavage;	*Akk* alleviated BW gain, fat mass, glucose tolerance, insulin sensitivity, and low-grade inflammation and reduced expression of genes associated with fatty acid synthesis and transport in both the liver and muscle.

[10] non-alcoholic steatohepatitis

N	Author, year, city, country	Disorder/ condition	Strain	Sex	Age	*AKK* strain	Diet	Intervention	Overall conclusion
31	Wu 2017, Hangzhou, China	Concanavalin A induced liver injury	SPF C57BL/6	M	4-5 w	live Muc^T	Standard diet	3×10^9 cfu; gavage daily for 2 w	*Akk* reduced BW, AST, ALT, liver damage and inflammation; reduced systemic inflammation; increased mucus thickness, and gut tight-junction proteins.
32	Rao 20121, Guangzhou, China	HFD-induced MAFLD[11]	C57BL/6	M	7-8 w	*Akk* (ATCC BAA-835)	HFD, 60% fat and 1.2% Cho for 17 w	1×10^8 cfu, gavage every other day at w 11 of the HFD/HC diet for 6 w	*Akk* reduced BW, hepatic steatosis, liver TG, TC, and L-aspartate, liver inflammation, and serum ALT, AST, ALP, TC, and LPS; increased microbiome α diversity, ilium Glut2, PGC1α, E-cadherin, mucus thickness, and villi length.
33	Abot 2023, Belgium	HFD-fed mice	C57BL/6J	M	9 w	Pasteurized *Akk* MucT	HFD	10^8 cfu; 12 w; oral gavage	*Akk* improved body weight, fat mass, insulin level, glycemia and glucose tolerance; modulated gut motility and decreased glucose absorption during a HFD consumption.
34	Ashrafian 2021, Tehran, Iran	Normal diet-fed mice	NR	NR	8 w	Live *Akk* MucT (ATCC BAA-835)<br>Pasteurized *Akk* MucT (ATCC BAA-835)	Standard normal diet	1×10^9 cfu; 5 w; oral gavage	Both Live and pasteurized *Akk* reduced BW, food intake, lipid profile, and serum inflammatory markers. Pasteurized *Akk* had more effect on inflammatory biomarkers improving intestinal integrity and reducing epithelial permeability.
35	Everard 2013, Brussels, Belgium	Obesity	C57BL/6	M	10 w	*Akk,* live<br>Heat-killed *Akk*	HFD	2×10^8 cfu; 4 w; oral gavage	*Akk* improved gut barrier function, metabolic endotoxemia, diet-induced obesity, and glucose homeostasis. Heat-killed *AKK* did not improve the metabolic profile or the mucus layer thickness.
36	Depommier 2020, Brussels, Belgium	Obesity	C57BL/6J	M	8 w	Pasteurized *Akk* (ATTC BAA-835)	HFD	2×10^8 cfu; oral gavage; 5 w	*Akk* modulated body composition, reduced TC, and increased energy expenditure
37	Ashrafian 2021 (2), Tehran, Iran	Obesity	C57BL/6	M	8 w	Live *Akk* MucT (ATCC BAA-835)<br>Pasteurized *Akk*<br>*Akk* EVs	HFD	1×10^9 cfu; 5 w<br>1×0^9 cfu; 5 w<br>10 µg; 5 w	*Akk* reduced BW, metabolic tissues weight, food consumption, lipid profile, inflammation, and blood glucose level. *Akk* and its EVs reduced liver, serum, and gut inflammation. All treatments improved gut microbiota composition and epithelial permeability.
38	Ashrafian 2019, Tehran, Iran	Obesity	C57BL/6	M	8 w	Live *Akk* MucT (ATCC BAA-835)<br>*Akk* EVs	HFD	1×10^9 cfu; 5 w<br>10 µg /200 µl; 5 w	*Akk* and EVs improved lipid profile, blood glucose level, and inflammation. EVs reduced body and fat mass weight more significantly than live bacterium.
39	Yura Choi 2021, Goyang-si, Korea	Obesity	C57BL6/N	M	6 w	Live *Akk* type strain BAA-835<br>Pasteurized *Akk* type strain BAA-835<br>Live *Akk* strain 19<br>Pasteurized *Akk* strain 19	HFD	1×10^8 cfu; 12 w (5 times/w); gavage	Both forms of intervention reduced BW, lipid profile, blood glucose, and inflammation and restored gut epithelial function and integrity. The pasteurized form was more potent in improving glucose tolerance than the live form.

[11] metabolic dysfunction-associated fatty liver disease

Table S2. SMD and 95% CI of the serum and gut inflammatory biomarkers (refers to Figure 2).

Included studies (n)	Effect sizes (n)	SMD (95% CI)	Outlier effect sizes (n)	SMD (95% CI) after removing outliers
**Gut TNFα**				
16	34	-1.15 (-1.67 to -0.63)	7	-0.92 (-1.28 to -0.57)
**Gut IL6**				
9	19	-1.40 (-2.28 to -0.51)	3	-0.60 (-1.04 to -0.16)
**Gut IL10**				
11	27	3.91 (1.42 to 6.39)	3	0.56 (0.28 to 0.84)
**Serum TNFα**				
6	9	-17.88 (-27.16 to -8.61)	1	-15.38 (-23.25 to -7.50)
**Serum IL6**				
5	8	-32.52 (-52.56 to -12.47)	No outlier effect size	
**Serum IL10**				
4	8	42.76 (-9.81 to 95.34)	1	17.35 (-2.47 to 37.17)
**Serum LPS**				
5	9	-2.94 (-5.90 to 0.01)	2	-2.62 (-5.39 to 0.14)

Table S3. Results of publication bias for studies measuring serum and gut inflammatory biomarkers according to funnel plots and Egger and Begg tests (refers to Figure 2).

Measured factor	Visual inspection of the funnel plot	Egger's test p.value	Begg's test p.value	Results
**Gut TNFα**	Slight evidence of publication bias	0.095	0.173	No significant small-study effects, indicating no strong evidence of publication
**Gut IL6**	Lack of publication bias	0.619	0.854	Lack of small-study effects and publication bias
**Gut IL10**	Slight evidence of publication bias	0.295	0.487	No significant small-study effects, indicating no strong evidence of publication
**Serum TNFα**	Slight evidence of publication bias	0.575	0.710	No significant small-study effects, indicating no strong evidence of publication bias
**Serum IL6**	Lack of publication bias	0.370	0.618	No significant small-study effects, indicating no strong evidence of publication bias
**Serum IL10**	Lack of publication bias	0.535	1.00	No significant small-study effects, indicating no strong evidence of publication bias
**Serum LPS**	Evidence of publication bias	0.001	0.707	Evidence of publication bias or small-study effects

Table S4. Sub-group analysis of the serum and gut inflammatory biomarkers for treatment type, dose, and duration (refers to Figure 2).

Subgroup		Included studies (n) [effect size (n)]	SMD (95% CI)	p value
**Gut TNFα**				
**Treatment type**	Live	8 [14]	-0.80 (-1.26 to -0.35)	<0.001
	Non-alive	5 [7]	-1.12 (-1.69 to -0.55)	<0.001
**Dose**	≤10^8 cfu	4 [12]	-0.57 (-0.84 to -0.29)	<0.001
	≥10^9 cfu	5 [6]	-0.88 (-1.71 to -0.06)	0.04
**Duration**	≤2 w	4 [6]	-1.09 (-1.98 to -0.19)	0.02
	3-6 w	4 [5]	-0.91 (-1.72 to -0.10)	0.03
	≥7 w	3 [10]	-0.70 (-1.00 to -0.40)	<0.001
**Gut IL6**				
**Treatment type**	Live	7 [12]	-0.44 (-0.80 to 0.08)	0.02
	Non-alive	1 [1]	-2.17 (-2.34 to -2.00)	<0.001
**Dose**	≤10^8 cfu	4 [8]	-0.13 (-0.22 to -0.03)	0.01
	≥10^9 cfu	3 [3]	-0.73 (-1.84 to 0.38)	0.20
**Duration**	≤2 w	4 [6]	-0.95 (-1.76 to -0.15)	0.02
	3-6 w	1 [1]	-0.08 (-0.88 to 0.72)	0.84
	≥7 w	2 [6]	-0.32 (-0.67 to 0.03)	0.08
**Gut IL10**				
**Treatment type**	Live	8 [13]	0.49 (0.18 to 0.80)	<0.001
	Non-alive	3 [8]	0.62 (0.09 to 1.14)	0.02
**Dose**	≤10^8 cfu	4 [10]	0.22 (-0.04 to 0.49)	0.10
	≥10^9 cfu	5 [7]	1.04 (0.29 to 1.79)	<0.001
**Duration**	≤2 w	5 [10]	0.83 (0.35 to 1.30)	<0.001
	3-6 w	2 [5]	0.69 (0.29 to 1.09)	<0.001
	≥7 w	2 [6]	0.11 (-0.31 to 0.52)	0.62
**Serum TNFα**				
**Treatment type**	Live	5 [5]	-9.86 (-18.37 to -1.34)	0.02
	Non-alive	2 [3]	-22.55 (-23.25 to -7.50)	<0.001
**Duration**	≤2 w	2 [2]	-6.13 (-25.27 to 13.01)	0.53
	3-6 w	2 [5]	-19.80 (-26.86 to -12.74)	<0.001
	≥7 w	1 [1]	-0.69 (-1.41 to 0.03)	0.06
**Serum IL-6**				
**Treatment type**	Live	5 [5]	-25.40 (-46.78 to -4.02)	0.02
	Non-alive	2 [3]	-43.86 (-86.39 to -1.33)	0.04
**Duration**	≤2 w	1 [1]	-3.84 (-11.4 to 3.72)	0.32
	3-6 w	2 [5]	-43.62 (-69.06 to -18.17)	<0.001
	≥7 w	2 [2]	-17.34 (-49.35 to 14.66)	0.29
**Serum IL-10**				
**Treatment type**	Live	4 [4]	7.49 (5.98 to 8.99)	<0.001
	Non-alive	2 [3]	28.28 (-16.81 to 73.36)	0.22
**Duration**	≤2 w	1 [1]	6.33 (-6.25 to 18.91)	0.32
	3-6 w	2 [4]	22.56 (-10.05 to 55.16)	0.18
	≥7 w	2 [2]	12.98 (2.37 to 23.58)	0.09

Table S5. SMD and 95% CI of gut health parameters (refers to Figure 3).

Included studies (n)	Effect sizes (n)	SMD (95% CI)	Outlier effect sizes (n)	SMD (95% CI) after removing outliers
**Colon length**				
9	19	1.03 (0.52 to 1.55)	3	0.93 (0.51 to 1.36)
**Mucus thickness**				
7	12	2.80 (1.04 to 4.56)	3	2.86 (1.69 to 4.03)
**ZO-1**				
11	25	0.93 (0.69 to 1.18)	4	0.73 (0.53 to 0.92)
**Occludin**				
13	29	1.48 (0.86 to 2.09)	3	1.06 (0.63 to 1.49)
**Claudin**				
12	24	2.13 (0.88 to 3.39)	1	1.24 (0.77 to 1.71)
**TL-R**				
5	13	2.15 (-0.26 to 4.57)	1	1.17 (-0.40 to 2.73)
**Muc-2**				
6	9	0.35 (-0.10 to 0.79)	1	0.11 (-0.12 to 0.35)

Table S6. Results of publication bias for studies measuring gut health parameters according to the funnel plots and Egger and Begg tests (refers to Figure 3).

Measured factor	Visual inspection of the funnel plot	Egger's test p.value	Begg's test p.value	Results
colon length	Slight evidence of publication bias	0.872	0.160	No significant small-study effects, indicating no strong evidence of publication
mucus thickness	Lack of publication bias	0.789	1.00	Lack of small-study effects and publication bias
ZO-1	Slight evidence of publication bias	0.372	0.109	No significant small-study effects, indicating no strong evidence of publication
Occludin	Slight evidence of publication bias	0.537	0.354	No significant small-study effects, indicating no strong evidence of publication
Claudin	Lack of publication bias	0.305	0.366	Lack of small-study effects and publication bias
TL-R	Lack of publication bias	0.587	1.00	Lack of small-study effects and publication bias
Muc-2	Slight evidence of publication bias	0.287	0.386	No significant small-study effects, indicating no strong evidence of publication

Table S7. Results of sub-group analysis of gut health parameters for treatment type, dose, and duration (refers to Figure 3).

Subgroup		Included studies (n) [effect size (n)]	SMD (95% CI)	p value
**Colon length**				
**Treatment type**	Live	6 [12]	0.71 (0.27 to 1.15)	<0.001
	Non-alive	2 [2]	1.48 (0.12 to 2.84)	0.03
**Dose**	≤10^8 cfu	4 [8]	0.76 (0.17 to 1.35)	0.01
	≥10^9 cfu	3 [5]	0.98 (0.27 to 1.70)	0.01
**Mucus thickness**				
**Treatment type**	Live	4 [6]	2.94 (0.79 to 5.08)	0.01
	Non-alive	2 [3]	2.73 (1.98 to 3.49)	<0.001
**Duration**	≤2 w	2 [3]	1.97 (-2.46 to 6.39)	0.38
	3-6 w	1 [1]	1.48 (-4.18 to 7.14)	0.61
	≥7 w	2 [5]	2.90 (1.89 to 3.91)	<0.001
**ZO-1**				
**Treatment type**	Live	10 [14]	0.60 (0.43 to 0.76)	<0.001
	Non-alive	5 [7]	0.76 (0.24 to 1.27)	<0.001
**Dose**	≤10^8 cfu	3 [9]	0.36 (0.31 to 0.42)	<0.001
	≥10^9 cfu	7 [9]	0.97 (0.72 to 1.23)	<0.001
**Duration**	≤2 w	2 [2]	0.66 (0.07 to 1.25)	0.03
	3-6 w	5 [8]	1.08 (0.77 to 1.39)	<0.001
	≥7 w	4 [11]	0.37 (0.31 to 0.44)	<0.001
**Occludin**				
**Treatment type**	Live	13 [18]	0.96 (0.50 to 1.42)	<0.001
	Non-alive	6 [8]	1.26 (0.31 to 2.22)	0.01
**Dose**	≤10^8 cfu	3 [9]	0.53 (0.21 to 0.48)	<0.001
	≥10^9 cfu	9 [11]	0.95 (0.38 to 1.52)	<0.001
**Duration**	≤2 w	4 [7]	1.23 (0.56 to 1.90)	<0.001
	3-6 w	6 [12]	1.38 (0.60 to 2.15)	<0.001
	≥7 w	3 [7]	0.24 (0.09 to 0.40)	<0.001
**Claudin**				
**Treatment type**	Live	11 [12]	1.12 (0.52 to 1.72)	<0.001
	Non-alive	6 [10]	1.37 (0.61 to 2.14)	<0.001
**Dose**	≤10^8 cfu	6 [9]	0.73 (-0.03 to 1.50)	0.06
	≥10^9 cfu	6 [7]	1.69 (0.95 to 2.44)	<0.001
**Duration**	≤2 w	4 [5]	1.20 (0.59 to 1.81)	<0.001
	3-6 w	5 [12]	1.66 (0.99 to 2.33)	<0.001
	≥7 w	2 [5]	0.33 (-0.12 to 0.59)	0.20
**TLR-2**				
**Treatment type**	Live	5 [6]	1.54 (-0.91 to 4.00)	0.22
	Non-alive	4 [6]	1.37 (0.61 to 2.14)	0.47

Table S8. SMD and 95% CI of glycemic control parameters, lipid profile, and liver health (refers to Figure 4).

Included studies (n)	Effect sizes (n)	SMD (95% CI)	Outlier effect sizes (n)	SMD (95% CI) after removing outliers
**Blood glucose**				
9	14	-12.14 (-18.54 to -5.73)	1	-10.06 (-15.69 to -4.43)
**Serum insulin**				
6	13	-2.56 (-4.59 to -0.53)	3	-0.40 (-0.71 to -0.08)
**HOMA.IR**				
3	7	-2.53 (-3.98 to -1.08)	1	-2.39 (-3.84 to -0.94)
**OGTT**				
5	12	-3495.96 (-5319.27 to -1672.65)	No outlier effect size	
**TG**				
13	27	-8.02 (-14.23 to -1.81)	3	-3.64 (-6.67 to -0.60)
**Cholesterol**				
11	24	-7.40 (-14.47 to -0.33)	2	-6.78 (-10.79 to -2.77)
**ALT**				
8	16	-38.86 (-53.87 to -23.84)	2	-36.14 (-50.66 to -21.62)
**AST**				
7	13	-46.08 (-68.65 to -23.51)	2	-42.87 (-65.56 to -20.17)

Table S9. Results of publication bias for studies measuring glycemic control parameters, lipid profile and liver health according to funnel plot and Egger and Begg tests (refers to Figure 4).

Measured factor	Visual inspection of the funnel plot	Egger's test p.value	Begg's test p.value	Results
blood glucose	Evidence of publication bias	0.001	0.176	Evidence of publication bias or small-study effects
Insulin	Evidence of publication bias	< 0.001	1.00	Evidence of publication bias or small-study effects
Homa.IR	Slight evidence of publication bias	0.077	0.180	No significant small-study effects, indicating no strong evidence of publication
TG	Evidence of publication bias	0.007	0.081	Evidence of publication bias or small-study effects
Cholesterol	Lack of publication bias	0.375	0.137	Lack of small-study effects and publication bias
ALT	Slight evidence of publication bias	0.106	0.546	No significant small-study effects, indicating no strong evidence of publication
AST	Lack of publication bias	0.989	0.137	No significant small-study effects, indicating no strong evidence of publication

Table S10. Results of sub-group analysis of glycemic control parameters, lipid profile and liver health for treatment type, dose, and duration (refers to Figure 4).

Subgroup		Included studies (n) [effect size (n)]	SMD (95% CI)	p value
**Blood glucose**				
**Treatment type**	Live	8 [9]	-9.10 (-15.39 to -2.80)	<0.001
	Non-alive	3 [4]	-11.55 (-26.96 to 0.86)	0.07
**Dose**	≤10^8 cfu	4 [4]	-8.67 (-20.79 to 3.45)	0.16
	≥10^9 cfu	5 [8]	-11.30 (-18.75 to -3.84)	<0.001
**Duration**	3-6 w	5 [8]	-14.14 (-21.47 to -6.80)	<0.001
	≥7 w	4 [5]	-1.88 (-3.55 to -0.21)	0.03
**ALT**				
**Treatment type**	Live	7 [9]	-35.28 (-55.52 to -15.04)	<0.001
	Non-alive	3 [5]	-38.04 (-60.23 to -15.85)	<0.001
**Dose**	≤10^8 cfu	3 [6]	-17.32 (-22.11 to -12.54)	<0.001
	≥10^9 cfu	4 [7]	-43.75 (-64.87 to -22.64)	<0.001
**Duration**	≤2 w	1 [2]	-32.02 (-47.09 to -16.95)	<0.001
	3-6 w	3 [6]	-48.66 (-74.98 to -22.35)	<0.001
	≥7 w	3 [6]	-23.46 (-38.59 to -8.33)	<0.001
**AST**				
**Treatment type**	Live	5 [6]	-34.30 (-64.15 to -4.45)	0.02
	Non-alive	3 [5]	-41.77 (-79.34 to -4.20)	<0.001
**Dose**	≤10^8 cfu	1 [4]	-10.11 (-15.86 to -4.37)	<0.001
	≥10^9 cfu	4 [6]	-56.51 (-84.70 to -28.32)	<0.001
**Duration**	3-6 w	2 [5]	-75.99 (-99.24 to -52.74)	<0.001
	≥7 w	3 [6]	-10.51 (-16.09 to -4.94)	<0.001
**TG**				
**Treatment type**	Live	10 [15]	-0.18 (-0.32 to -0.03)	0.02
	Non-alive	6 [8]	-5.41 (-12.96 to 2.13)	0.16
**Dose**	≤10^8 cfu	6 [12]	0.04 (-2.12 to 2.19)	0.97
	≥10^9 cfu	6 [9]	-9.01 (-14.83 to -3.18)	<0.001
**Duration**	≤2 w	1 [2]	-8.39 (-14.90 to -1.87)	0.01
	3-6 w	7 [10]	-7.30 (-12.38 to -2.22)	<0.001
	≥7 w	5 [11]	0.27 (-1.77 to 2.30)	0.80
**Cholesterol**				
**Treatment type**	Live	10 [14]	-4.34 (-8.48 to -0.19)	0.04
	Non-alive	4 [6]	-12.55 (-20.72 to -4.39)	<0.001
**Dose**	≤10^8 cfu	6 [10]	-1.13 (-1.60 to -0.66)	<0.001
	≥10^9 cfu	5 [8]	-7.97 (-16.23 to 0.29)	0.06
**Duration**	3-6 w	6 [11]	-10.52 (-16.86 to -4.18)	<0.001
	≥7 w	5 [9]	-1.09 (-1.42 to -0.76)	<0.001

Table S11. SMD and 95% CI of body weight for studies of GI and metabolic disorders (refers to Figure 5).

Included studies (n)	Effect sizes (n)	SMD (95% CI)	Outlier effect sizes (n)	SMD (95% CI) after removing outliers
**Body weight of mice with GI disorders**				
4	7	1.77 (0.32 to 3.32)	No outlier effect size	
**Body weight of mice with metabolic disorders**				
12	26	-3.07 (-3.97 to -2.17)	3	-2.50 (-3.15 to -1.86)

Table S12. Results of publication bias for studies measuring body weight according to funnel plots and Egger and Begg tests (refers to Figure 5).

Measured factor	Visual inspection of the funnel plot	Egger's test p.value	Begg's test p.value	Results
Body weight (GI disorders)	Slight evidence of publication bias	0.538	0.163	No significant small-study effects, indicating no strong evidence of publication bias
Body weight (metabolic disorders)	Slight evidence of publication bias	1.00	0.480	No significant small-study effects, indicating no strong evidence of publication bias

Table S13. Results of sub-group analysis of body weight for treatment type, dose, and duration (refers to Figure 5).

Subgroup		Included studies (n) [effect size (n)]	SMD (95% CI)	p value
**Body weight (GI disorders)**				
**Dose**	$\leq 10^8$ cfu	2 [2]	2.86 (2.17 to 3.55)	<0.001
	$\geq 10^9$ cfu	2 [5]	1.30 (-0.74 to 3.34)	<0.21
**Body weight (metabolic disorders)**				
**Treatment type**	Live	7 [11]	-2.41 (-3.37 to -1.46)	<0.001
	Non-alive	7 [11]	-2.61 (-3.53 to -1.69)	<0.001
**Dose**	$\leq 10^8$ cfu	6 [13]	-3.19 (-4.05 to -2.33)	<0.001
	$\geq 10^9$ cfu	4 [6]	-1.60 (-2.64 to -0.57)	<0.001
**Duration**	3-6 w	6 [15]	-1.57 (-2.04 to -1.09)	<0.001
	≥7 w	3 [7]	-4.14 (-4.53 to -3.74)	<0.001

Table S14. Search strategy for *Dedulfovibrio* species.

- ***g_Desulfovibrio* taxonomy search (BLASTn)**

ASVs ID	Blast results	Query cover	Percent identity
a2df8b2c47a367afb9aefb636b45f048	Desulfovibrio sp. strain 16x 16S ribosomal RNA gene, partial sequence	100%	100%
8e4015c1fbee5ee49dda8213fb96ffc1	Desulfovibrio sp. strain 16x 16S ribosomal RNA gene, partial sequence	100%	100%
06cd324dcbdb96bdc36a5a2f2c158bb5	Desulfovibrio sp. strain 16x 16S ribosomal RNA gene, partial sequence	100%	99.77%
7f9502d28125a5f6deda27a4237c5111	Desulfovibrio sp. strain 148x 16S ribosomal RNA gene, partial sequence	100%	99.77%
cfad5e9a4368d32268ec7bbcc060783a	Desulfovibrio sp. strain 16x 16S ribosomal RNA gene, partial sequence	100%	99.77%
f782e5aec5302ad71c18379b0ce30d19	Desulfovibrio sp. strain 148x 16S ribosomal RNA gene, partial sequence,<br>Desulfovibrio sp. ABHU2SB 16S ribosomal RNA gene, partial sequence	100%	100%
8bd76b9b965a0b5041c2b67fc6eb24c5	Desulfovibrio sp. strain 148x 16S ribosomal RNA gene, partial sequence	100%	99.55%
037fd3ff790a3d7306864a8657e0a1b8	Desulfovibrio sp. strain 16x 16S ribosomal RNA gene, partial sequence	100%	100%
f56c270199244e875035bc529ba05943	Uncultured Desulfovibrio sp. clone OTU393 16S ribosomal RNA gene, partial sequence	100%	100%
b57974fa6fe9bf79765deeaaf6857630	Desulfovibrio sp. strain 16x 16S ribosomal RNA gene, partial sequence	100%	100%
774f67d6c295ddd7e3725d32e601dd4a	Desulfovibrio sp. strain 148x 16S ribosomal RNA gene, partial sequence	100%	100%
c37a1c2769b5d8d065cd943621881407	Uncultured bacterium clone 10821 16S ribosomal RNA gene, partial sequence	100%	100%
a2731aabe0dd79e8775328f7e10d21f8	Desulfovibrio sp. strain 16x 16S ribosomal RNA gene, partial sequence	100%	99.76%
59e69e23ced2cfa097044f97c66cfd26	Uncultured bacterium clone R-8230 16S ribosomal RNA gene, partial sequence	100%	100%
4579440529ccfdf0ed8fb8ea86dc7a5e	Uncultured Desulfovibrio sp. clone OTU393 16S ribosomal RNA gene, partial sequence	97%	100%
c3e7fccb2c7b8e24b40260c2d55c4ed5	Desulfovibrio sp. strain 16x 16S ribosomal RNA gene, partial sequence	100%	100%
31cc312016596a9b9f140bb80b56d2ac	Desulfovibrio sp. wp13 16S ribosomal RNA gene, partial sequence	100%	92.19%
abb243249a27ae65302d6535832a4f51	Desulfovibrio sp. ABHU1SBfatS 16S ribosomal RNA gene, partial sequence	100%	98.28%
f8fe7560968612df736a0362242afcc0	Desulfovibrio sp. strain 148x 16S ribosomal RNA gene, partial sequence	100%	99.77%
449efe9cddfe6738348eb0c929ca87b5	Uncultured Proteobacteria bacterium partial 16S rRNA gene, isolate OTU1144	100%	94.49%
452c8d71bd468414fe8fd69f3ec940fc	Desulfovibrio sp. strain 16x 16S ribosomal RNA gene, partial sequence	100%	97.73%
35d7adb74c993c8f901b01fdadc9be76	Uncultured bacterium clone denovo2942_N27_151186 16S ribosomal RNA gene, partial sequence	100%	99.74%
bd80c9eb2e292527af0e738de4b38b37	Uncultured Desulfovibrio sp. clone OTU_1082 16S ribosomal RNA gene, partial sequence	100%	100%
af87309cc69c2ac9ebdf7d7d77fd6c66	Uncultured bacterium clone 16S(V3-V4)-831 16S ribosomal RNA gene, partial sequence	99%	92.37%
618808eb71e2853040d99990de6117d9	Desulfovibrio sp. strain 148x 16S ribosomal RNA gene, partial sequence	100%	99.76%
279a00310e2559013199c91b7554d6dc	Uncultured prokaryote gene for 16S ribosomal RNA, partial sequence, OTU:AA6221	100%	91.84%
d7843f20876d420d578bcc095e51e2aa	Uncultured Desulfovibrio sp. clone 913 16S ribosomal RNA gene, partial sequence	99%	92.99%
41366c162a4f76898bd49cb583ac9dd1	Uncultured bacterium clone denovo29974_N12_2834 16S ribosomal RNA gene, partial sequence	99%	92.29%
fc54d2980c02371a8fc73926b120d06b	Uncultured Proteobacteria bacterium partial 16S rRNA gene, isolate OTU1144	99%	94.90%

- No species were identified via the BLASTn search.

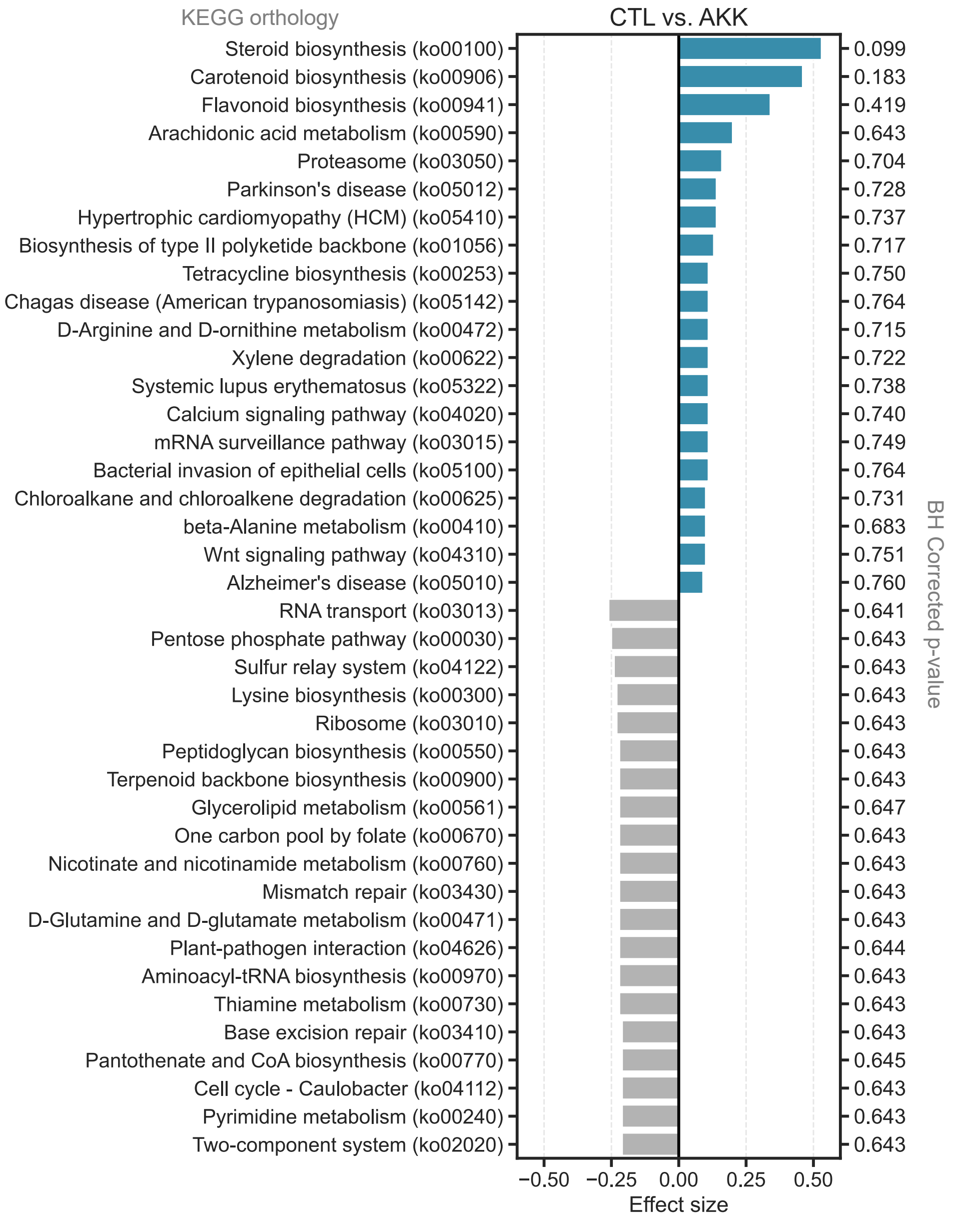

Figure S1. Differences in predicted KEGG pathway between CTL and AKK groups. ALDEx2 was used to measure the differences between CTL and AKK groups. Significance was determined using Welch's t-test, and p-values were adjusted for false discovery rate (FDR) using the Benjamini-Hochberg adjustment.

Hint: Supplement type sub-groups (alive = 1, non-alive = 2), Dose subgroups ($\leq 10^8 = 1$, $\geq 10^9 = 2$), Duration (≤2weeks= 1, 3-6 weeks = 2, ≥7weeks = 3)

Gut inflammation

TNFα

Supplement type

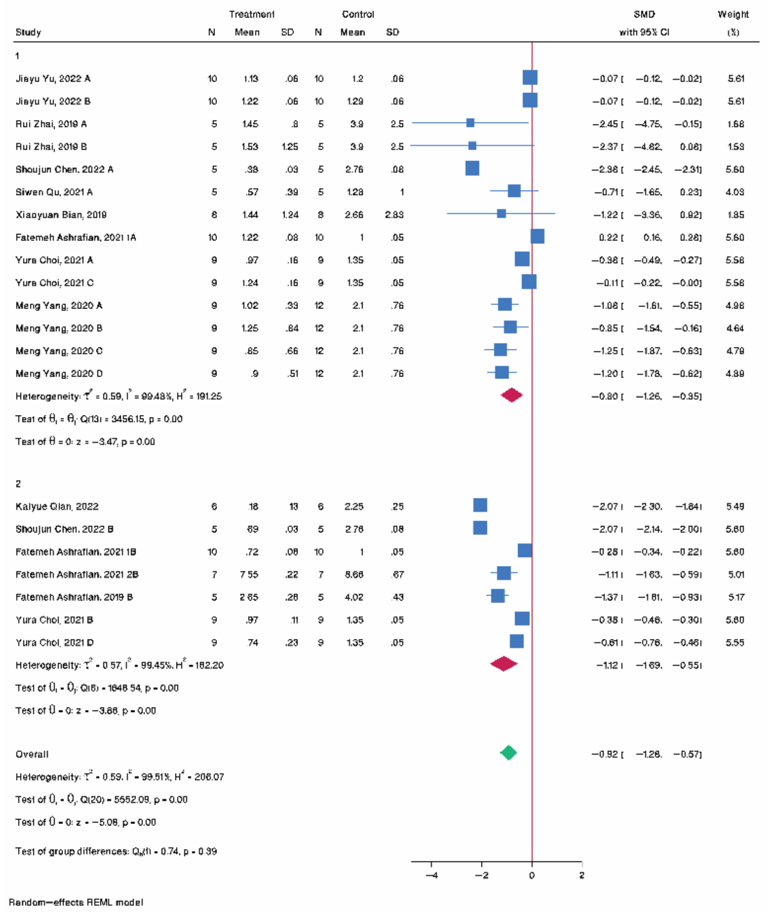

Dose

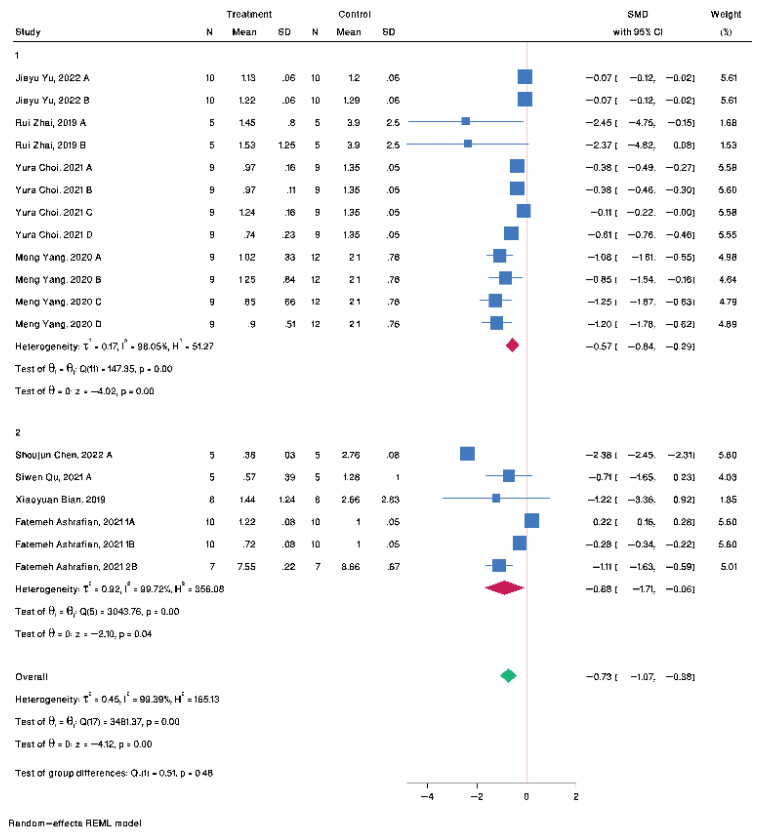

Duration

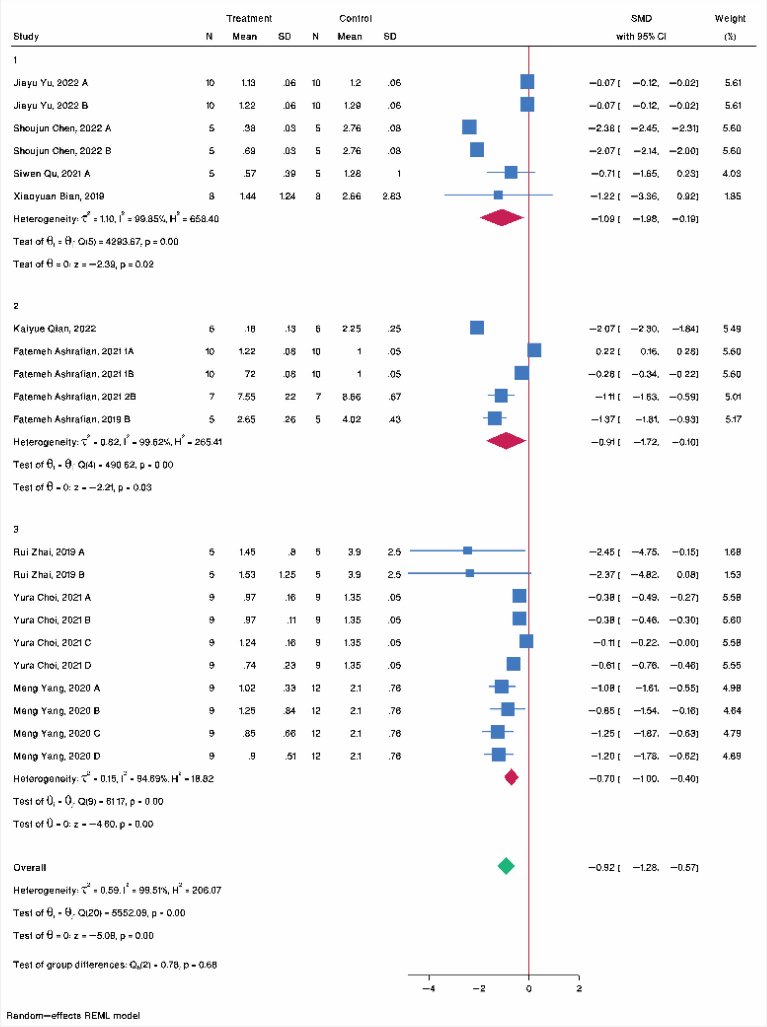
Treatment
Control
SMD with 95% CI
Weight (%)
Study N Mean SD N Mean SD
1
Jiayu Yu, 2022 A 10 1.13 .06 10 1.2 .06 −0.07 [−0.12, −0.02] 5.61
Jiayu Yu, 2022 B 10 1.22 .06 10 1.29 .06 −0.07 [−0.12, −0.02] 5.61
Shoujun Chen, 2022 A 5 .38 .03 5 2.76 .08 −2.38 [−2.45, −2.31] 5.60
Shoujun Chen, 2022 B 5 .69 .03 5 2.76 .08 −2.07 [−2.14, −2.00] 5.60
Siwen Qu, 2021 A 5 .57 .39 5 1.28 1 −0.71 [−1.65, 0.23] 4.03
Xiaoyuan Bian, 2019 8 1.44 1.24 8 2.66 2.83 −1.22 [−3.36, 0.92] 1.85
Heterogeneity: τ² = 1.10, I² = 99.85%, H² = 658.40 −1.09 [−1.98, −0.19]
Test of θi = θj: Q(5) = 4293.67, p = 0.00
Test of θ = 0: z = −2.39, p = 0.02
2
Kaiyue Qian, 2022 6 .18 .13 6 2.25 .25 −2.07 [−2.30, −1.84] 5.49
Fatemeh Ashrafian, 2021 1A 10 1.22 .08 10 1 .05 0.22 [0.16, 0.28] 5.60
Fatemeh Ashrafian, 2021 1B 10 .72 .08 10 1 .05 −0.28 [−0.34, −0.22] 5.60
Fatemeh Ashrafian, 2021 2B 7 7.55 .22 7 8.66 .67 −1.11 [−1.63, −0.59] 5.01
Fatemeh Ashrafian, 2019 B 5 2.65 .26 5 4.02 .43 −1.37 [−1.81, −0.93] 5.17
Heterogeneity: τ² = 0.82, I² = 99.62%, H² = 265.41 −0.91 [−1.72, −0.10]
Test of θi = θj: Q(4) = 490.62, p = 0.00
Test of θ = 0: z = −2.21, p = 0.03
3
Rui Zhai, 2019 A 5 1.45 .8 5 3.9 2.5 −2.45 [−4.75, −0.15] 1.68
Rui Zhai, 2019 B 5 1.53 1.25 5 3.9 2.5 −2.37 [−4.82, 0.08] 1.53
Yura Choi, 2021 A 9 .97 .16 9 1.35 .05 −0.38 [−0.49, −0.27] 5.58
Yura Choi, 2021 B 9 .97 .11 9 1.35 .05 −0.38 [−0.46, −0.30] 5.60
Yura Choi, 2021 C 9 1.24 .16 9 1.35 .05 −0.11 [−0.22, −0.00] 5.58
Yura Choi, 2021 D 9 .74 .23 9 1.35 .05 −0.61 [−0.76, −0.46] 5.55
Meng Yang, 2020 A 9 1.02 .33 12 2.1 .76 −1.08 [−1.61, −0.55] 4.98
Meng Yang, 2020 B 9 1.25 .84 12 2.1 .76 −0.85 [−1.54, −0.16] 4.64
Meng Yang, 2020 C 9 .85 .66 12 2.1 .76 −1.25 [−1.87, −0.63] 4.79
Meng Yang, 2020 D 9 .9 .51 12 2.1 .76 −1.20 [−1.78, −0.62] 4.89
Heterogeneity: τ² = 0.15, I² = 94.69%, H² = 18.82 −0.70 [−1.00, −0.40]
Test of θi = θj: Q(9) = 61.17, p = 0.00
Test of θ = 0: z = −4.60, p = 0.00
Overall −0.92 [−1.28, −0.57]
Heterogeneity: τ² = 0.59, I² = 99.51%, H² = 206.07
Test of θi = θj: Q(20) = 5552.09, p = 0.00
Test of θ = 0: z = −5.08, p = 0.00
Test of group differences: Qb(2) = 0.78, p = 0.68
−4
−2
0
2
Random−effects REML model

IL-6

Supplement type

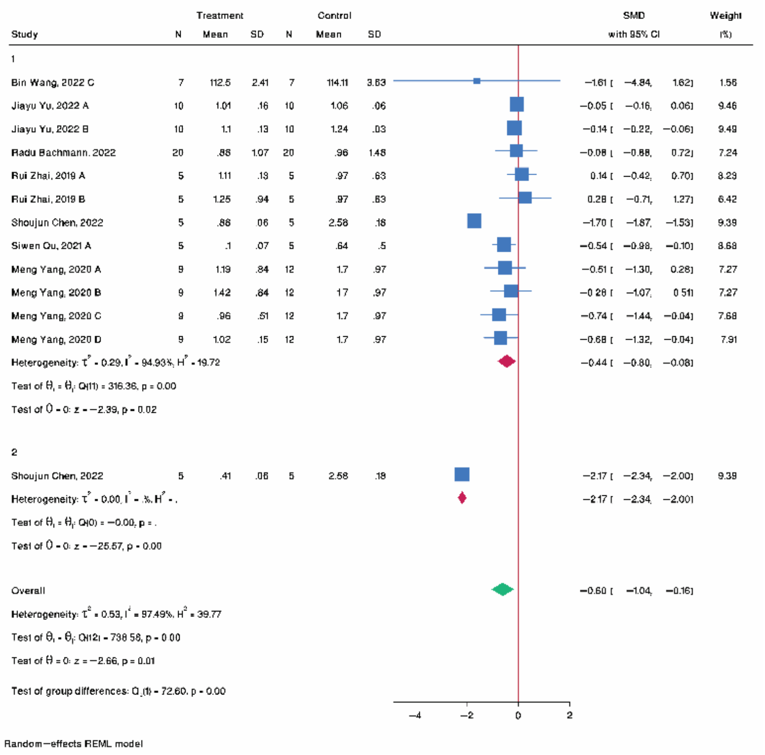

Dose

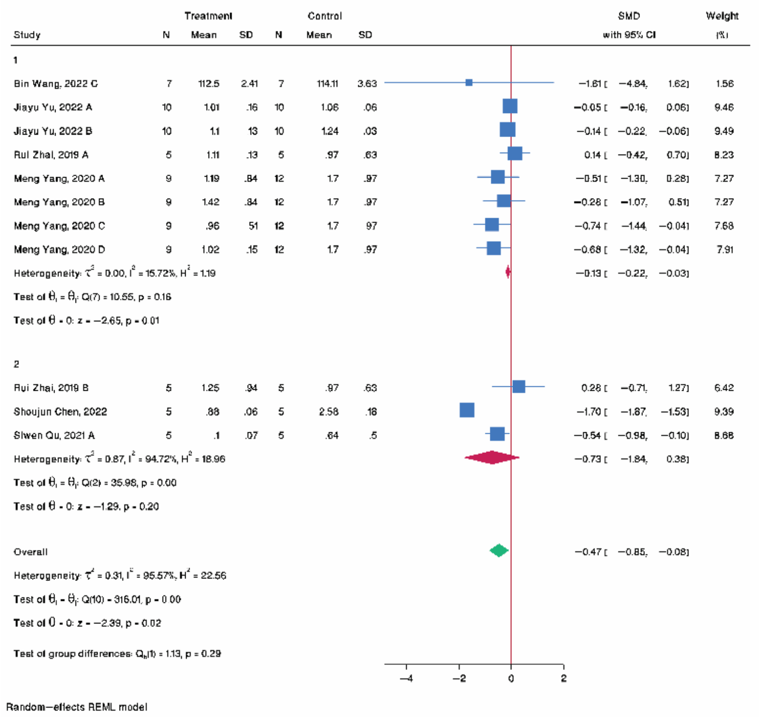

Duration

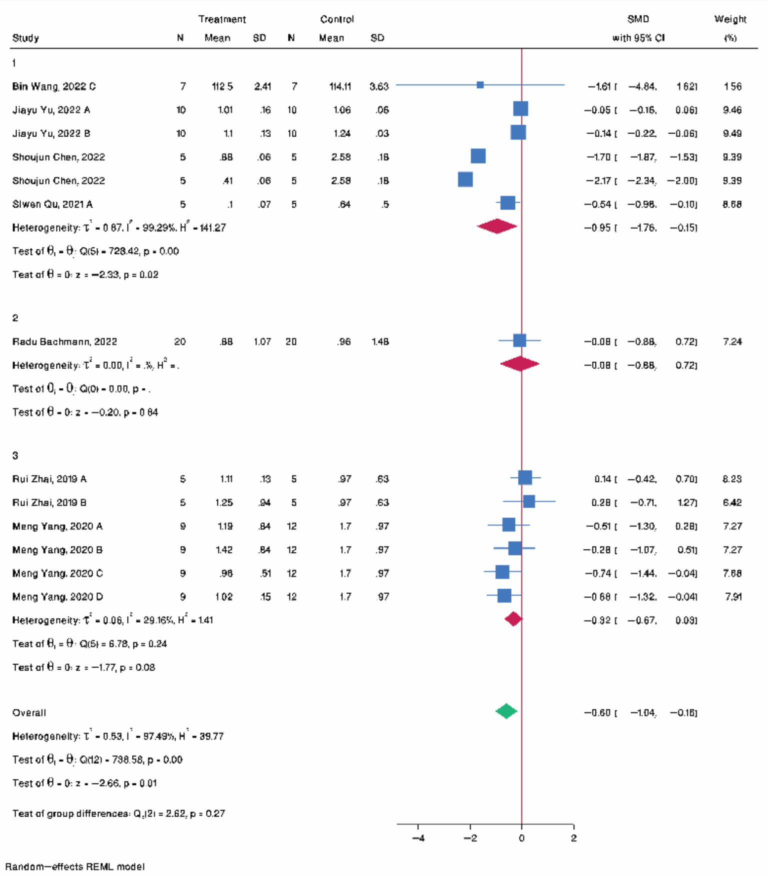

Treatment
Control
SMD with 95% CI
Weight (%)
Study N Mean SD N Mean SD
1
Bin Wang, 2022 C 7 112.5 2.41 7 114.11 3.63 −1.61 [−4.84, 1.62] 1.56
Jiayu Yu, 2022 A 10 1.01 .16 10 1.06 .06 −0.05 [−0.16, 0.06] 9.46
Jiayu Yu, 2022 B 10 1.1 .13 10 1.24 .03 −0.14 [−0.22, −0.06] 9.49
Shoujun Chen, 2022 5 .88 .06 5 2.58 .18 −1.70 [−1.87, −1.53] 9.39
Shoujun Chen, 2022 5 .41 .06 5 2.58 .18 −2.17 [−2.34, −2.00] 9.39
Siwen Qu, 2021 A 5 .1 .07 5 .64 .5 −0.54 [−0.98, −0.10] 8.68
Heterogeneity: τ² = 0.87, I² = 99.29%, H² = 141.27 −0.95 [−1.76, −0.15]
Test of θi = θj: Q(5) = 728.42, p = 0.00
Test of θ = 0: z = −2.33, p = 0.02
2
Radu Bachmann, 2022 20 .88 1.07 20 .96 1.48 −0.08 [−0.88, 0.72] 7.24
Heterogeneity: τ² = 0.00, I² = .%, H² = . −0.08 [−0.88, 0.72]
Test of θi = θj: Q(0) = 0.00, p = .
Test of θ = 0: z = −0.20, p = 0.84
3
Rui Zhai, 2019 A 5 1.11 .13 5 .97 .63 0.14 [−0.42, 0.70] 8.23
Rui Zhai, 2019 B 5 1.25 .94 5 .97 .63 0.28 [−0.71, 1.27] 6.42
Meng Yang, 2020 A 9 1.19 .84 12 1.7 .97 −0.51 [−1.30, 0.28] 7.27
Meng Yang, 2020 B 9 1.42 .84 12 1.7 .97 −0.28 [−1.07, 0.51] 7.27
Meng Yang, 2020 C 9 .98 .51 12 1.7 .97 −0.74 [−1.44, −0.04] 7.68
Meng Yang, 2020 D 9 1.02 .15 12 1.7 .97 −0.68 [−1.32, −0.04] 7.91
Heterogeneity: τ² = 0.06, I² = 29.16%, H² = 1.41 −0.32 [−0.67, 0.03]
Test of θi = θj: Q(5) = 6.78, p = 0.24
Test of θ = 0: z = −1.77, p = 0.08
Overall −0.60 [−1.04, −0.16]
Heterogeneity: τ² = 0.53, I² = 97.49%, H² = 39.77
Test of θi = θj: Q(12) = 738.58, p = 0.00
Test of θ = 0: z = −2.66, p = 0.01
Test of group differences: Qb(2) = 2.62, p = 0.27
−4
−2
0
2
Random−effects REML model

IL-10

Supplement type

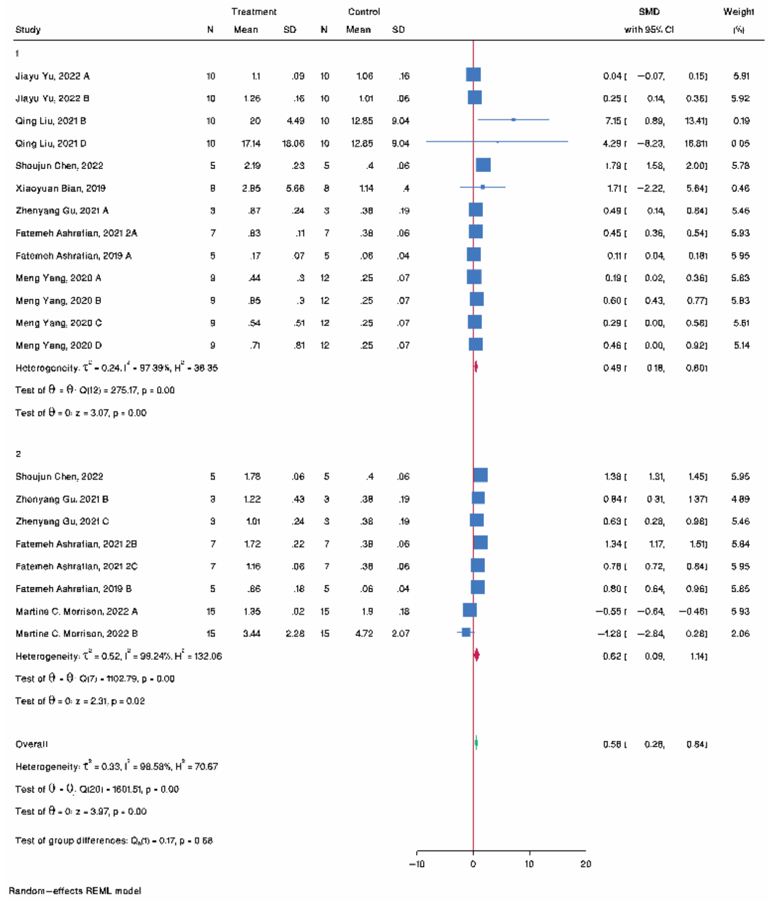

Dose

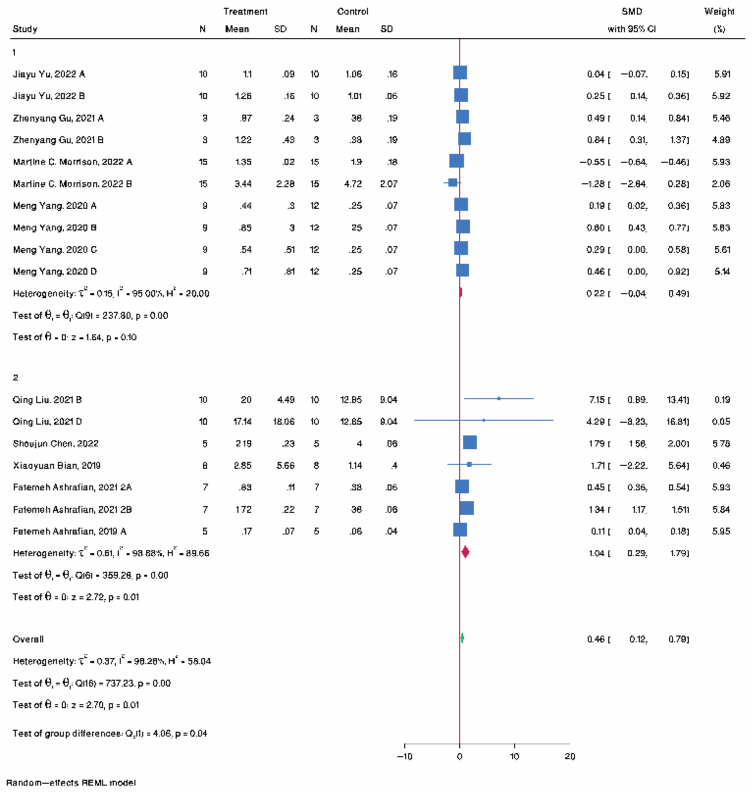

Duration

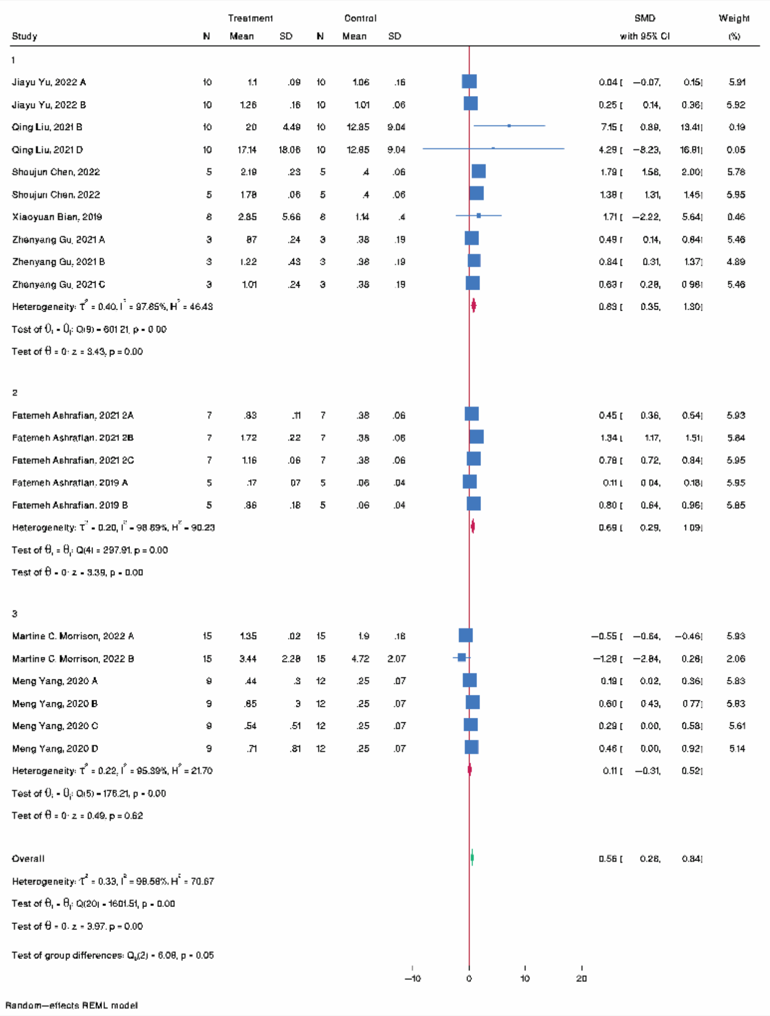
Treatment
Control
SMD with 95% CI
Weight (%)
Study N Mean SD N Mean SD
1
Jiayu Yu, 2022 A 10 1.1 .09 10 1.06 .16 0.04 [−0.07, 0.15] 5.91
Jiayu Yu, 2022 B 10 1.26 .16 10 1.01 .06 0.25 [0.14, 0.36] 5.92
Qing Liu, 2021 B 10 20 4.49 10 12.85 9.04 7.15 [0.89, 13.41] 0.19
Qing Liu, 2021 D 10 17.14 18.06 10 12.85 9.04 4.29 [−8.23, 16.81] 0.05
Shoujun Chen, 2022 5 2.19 .23 5 .4 .06 1.79 [1.58, 2.00] 5.78
Shoujun Chen, 2022 5 1.78 .06 5 .4 .06 1.38 [1.31, 1.45] 5.95
Xiaoyuan Bian, 2019 8 2.85 5.66 8 1.14 .4 1.71 [−2.22, 5.64] 0.46
Zhenyang Gu, 2021 A 3 .87 .24 3 .38 .19 0.49 [0.14, 0.84] 5.46
Zhenyang Gu, 2021 B 3 1.22 .43 3 .38 .19 0.84 [0.31, 1.37] 4.89
Zhenyang Gu, 2021 C 3 1.01 .24 3 .38 .19 0.63 [0.28, 0.98] 5.46
Heterogeneity: τ² = 0.40, I² = 97.85%, H² = 46.43 0.83 [0.35, 1.30]
Test of θi = θj: Q(9) = 601.21, p = 0.00
Test of θ = 0: z = 3.43, p = 0.00
2
Fatemeh Ashrafian, 2021 2A 7 .83 .11 7 .38 .06 0.45 [0.36, 0.54] 5.93
Fatemeh Ashrafian, 2021 2B 7 1.72 .22 7 .38 .06 1.34 [1.17, 1.51] 5.84
Fatemeh Ashrafian, 2021 2C 7 1.16 .06 7 .38 .06 0.78 [0.72, 0.84] 5.95
Fatemeh Ashrafian, 2019 A 5 .17 .07 5 .06 .04 0.11 [0.04, 0.18] 5.95
Fatemeh Ashrafian, 2019 B 5 .86 .18 5 .06 .04 0.80 [0.64, 0.96] 5.85
Heterogeneity: τ² = 0.20, I² = 98.89%, H² = 90.23 0.69 [0.29, 1.09]
Test of θi = θj: Q(4) = 297.91, p = 0.00
Test of θ = 0: z = 3.39, p = 0.00
3
Martine C. Morrison, 2022 A 15 1.35 .02 15 1.9 .18 −0.55 [−0.64, −0.46] 5.93
Martine C. Morrison, 2022 B 15 3.44 2.28 15 4.72 2.07 −1.28 [−2.84, 0.28] 2.06
Meng Yang, 2020 A 9 .44 .3 12 .25 .07 0.19 [0.02, 0.36] 5.83
Meng Yang, 2020 B 9 .85 .3 12 .25 .07 0.60 [0.43, 0.77] 5.83
Meng Yang, 2020 C 9 .54 .51 12 .25 .07 0.29 [0.00, 0.58] 5.61
Meng Yang, 2020 D 9 .71 .81 12 .25 .07 0.46 [0.00, 0.92] 5.14
Heterogeneity: τ² = 0.22, I² = 95.39%, H² = 21.70 0.11 [−0.31, 0.52]
Test of θi = θj: Q(5) = 176.21, p = 0.00
Test of θ = 0: z = 0.49, p = 0.62
Overall 0.56 [0.28, 0.84]
Heterogeneity: τ² = 0.33, I² = 98.58%, H² = 70.67
Test of θi = θj: Q(20) = 1601.51, p = 0.00
Test of θ = 0: z = 3.97, p = 0.00
Test of group differences: Qb(2) = 6.08, p = 0.05
−10
0
10
20
Random−effects REML model

Serum inflammation

TNFα

Supplement type

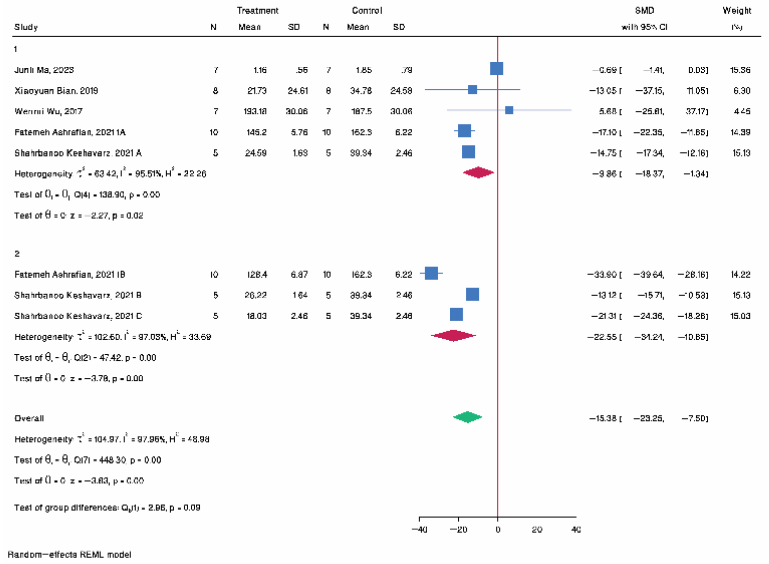

Duration

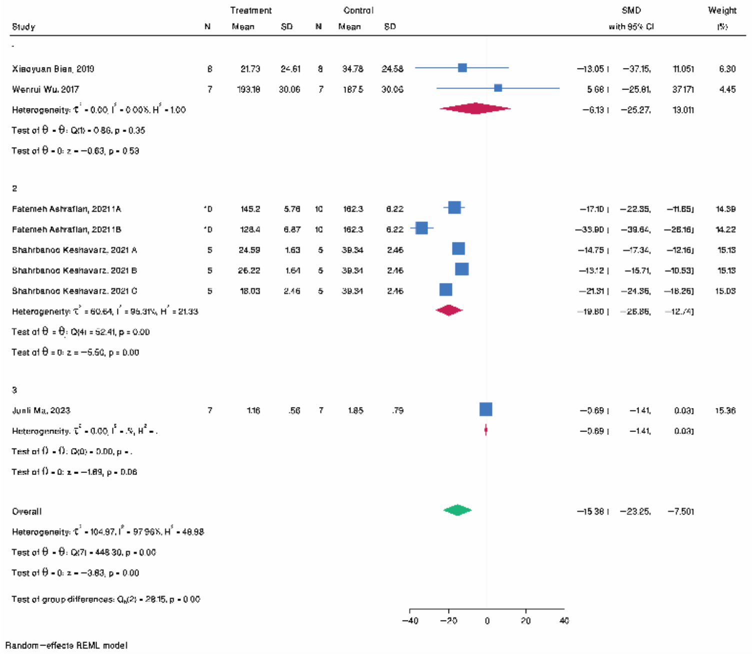

IL-6

Supplement type

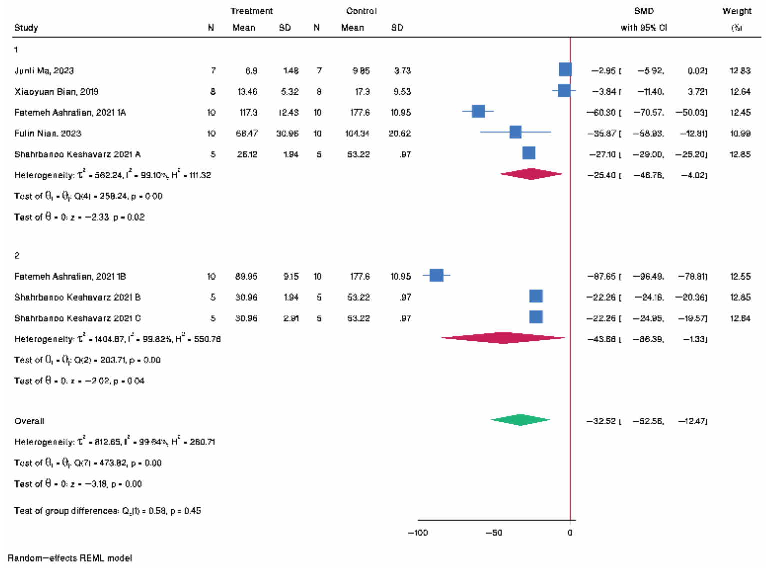

Duration

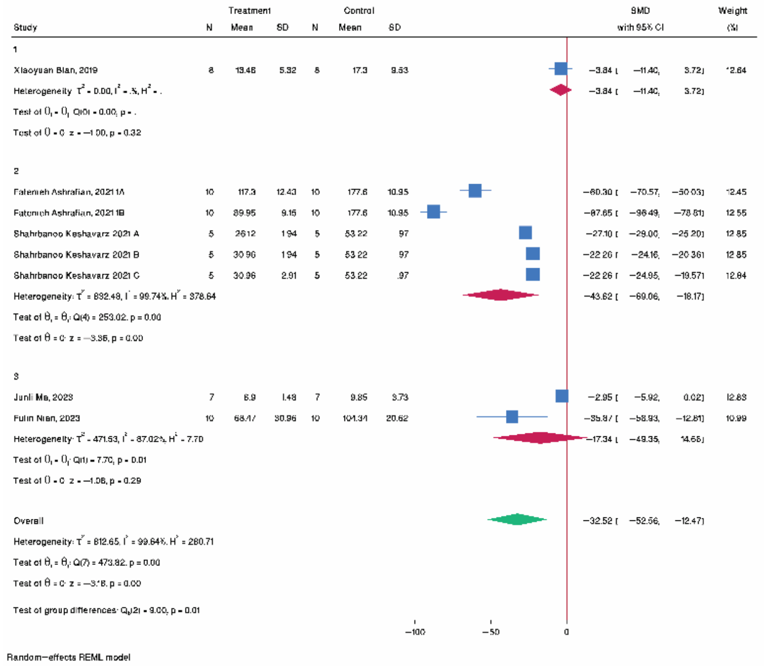

IL-10

Supplement type

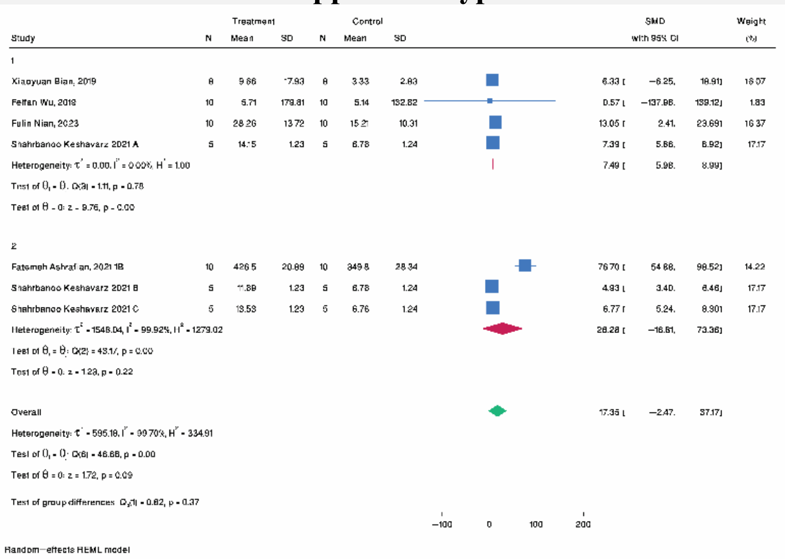

Gut barrier health

ZO-1

Supplement type

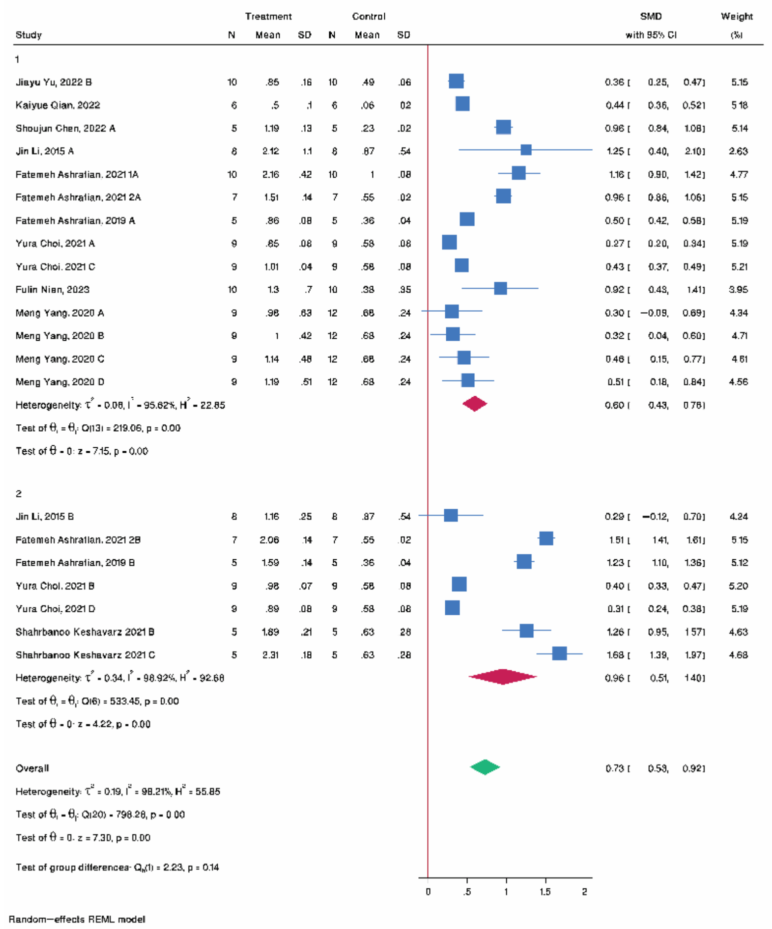

Dose

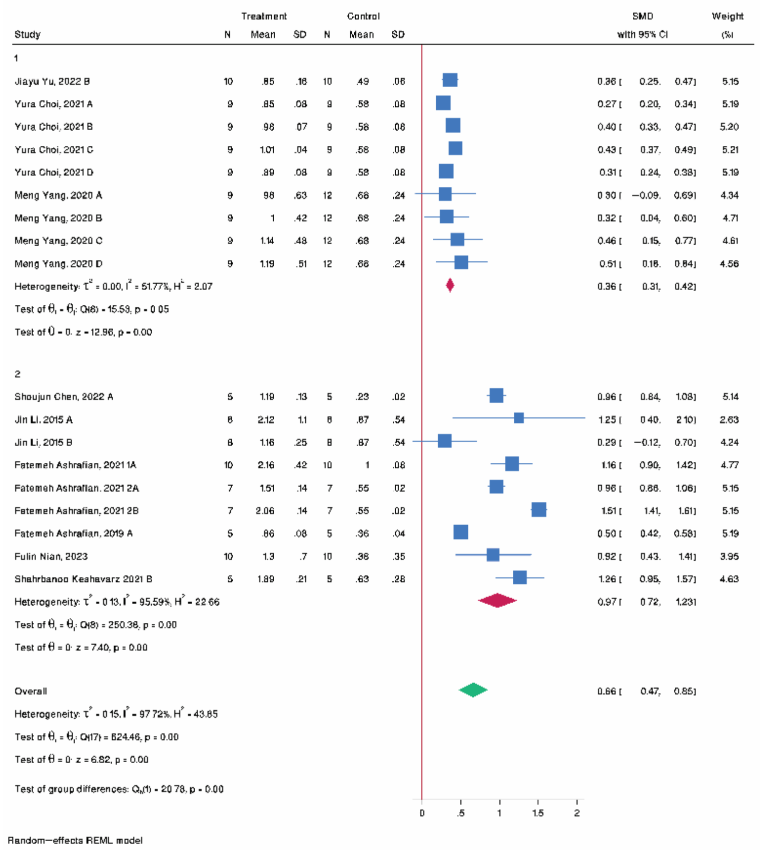

Duration

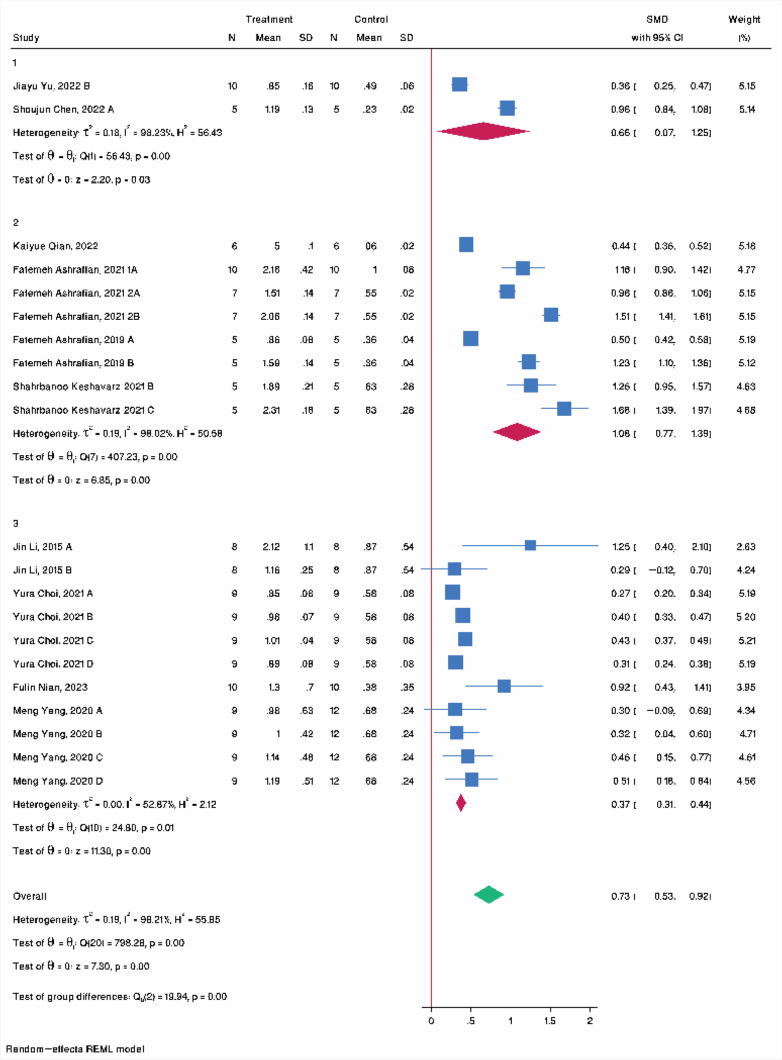

Treatment
Control
SMD with 95% CI
Weight (%)
Study N Mean SD N Mean SD
1
Jiayu Yu, 2022 B 10 .85 .16 10 .49 .06 0.36 [0.25, 0.47] 5.15
Shoujun Chen, 2022 A 5 1.19 .13 5 .23 .02 0.96 [0.84, 1.08] 5.14
Heterogeneity: τ² = 0.18, I² = 98.23%, H² = 56.43 0.66 [0.07, 1.25]
Test of θᵢ = θⱼ: Q(1) = 56.43, p = 0.00
Test of θ = 0: z = 2.20, p = 0.03
2
Kaiyue Qian, 2022 6 .5 .1 6 .06 .02 0.44 [0.36, 0.52] 5.18
Fatemeh Ashrafian, 2021 1A 10 2.18 .42 10 1 .08 1.16 [0.90, 1.42] 4.77
Fatemeh Ashrafian, 2021 2A 7 1.51 .14 7 .55 .02 0.96 [0.86, 1.06] 5.15
Fatemeh Ashrafian, 2021 2B 7 2.06 .14 7 .55 .02 1.51 [1.41, 1.61] 5.15
Fatemeh Ashrafian, 2019 A 5 .86 .08 5 .36 .04 0.50 [0.42, 0.58] 5.19
Fatemeh Ashrafian, 2019 B 5 1.59 .14 5 .36 .04 1.23 [1.10, 1.36] 5.12
Shahrbanoo Keshavarz 2021 B 5 1.89 .21 5 .63 .28 1.26 [0.95, 1.57] 4.63
Shahrbanoo Keshavarz 2021 C 5 2.31 .18 5 .63 .28 1.68 [1.39, 1.97] 4.68
Heterogeneity: τ² = 0.19, I² = 98.02%, H² = 50.58 1.08 [0.77, 1.39]
Test of θᵢ = θⱼ: Q(7) = 407.23, p = 0.00
Test of θ = 0: z = 6.85, p = 0.00
3
Jin Li, 2015 A 8 2.12 1.1 8 .87 .54 1.25 [0.40, 2.10] 2.63
Jin Li, 2015 B 8 1.16 .25 8 .87 .54 0.29 [−0.12, 0.70] 4.24
Yura Choi, 2021 A 9 .85 .06 9 .58 .08 0.27 [0.20, 0.34] 5.19
Yura Choi, 2021 B 9 .98 .07 9 .58 .08 0.40 [0.33, 0.47] 5.20
Yura Choi, 2021 C 9 1.01 .04 9 .58 .08 0.43 [0.37, 0.49] 5.21
Yura Choi, 2021 D 9 .89 .06 9 .58 .08 0.31 [0.24, 0.38] 5.19
Fulin Nian, 2023 10 1.3 .7 10 .38 .35 0.92 [0.43, 1.41] 3.95
Meng Yang, 2020 A 9 .98 .63 12 .68 .24 0.30 [−0.09, 0.69] 4.34
Meng Yang, 2020 B 9 1 .42 12 .68 .24 0.32 [0.04, 0.60] 4.71
Meng Yang, 2020 C 9 1.14 .48 12 .68 .24 0.46 [0.15, 0.77] 4.61
Meng Yang, 2020 D 9 1.19 .51 12 .68 .24 0.51 [0.18, 0.84] 4.56
Heterogeneity: τ² = 0.00, I² = 52.67%, H² = 2.12 0.37 [0.31, 0.44]
Test of θᵢ = θⱼ: Q(10) = 24.80, p = 0.01
Test of θ = 0: z = 11.30, p = 0.00
Overall 0.73 [0.53, 0.92]
Heterogeneity: τ² = 0.19, I² = 98.21%, H² = 55.85
Test of θᵢ = θⱼ: Q(20) = 798.28, p = 0.00
Test of θ = 0: z = 7.30, p = 0.00
Test of group differences: Qb(2) = 19.94, p = 0.00
0 .5 1 1.5 2
Random−effects REML model

Claudin

Supplement type

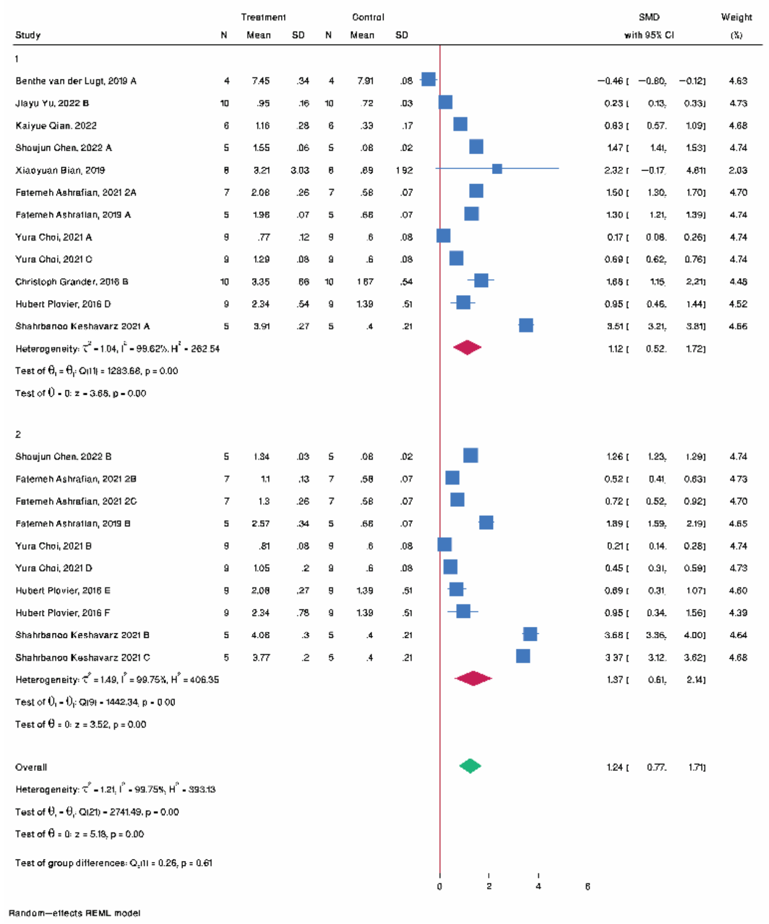

Dose

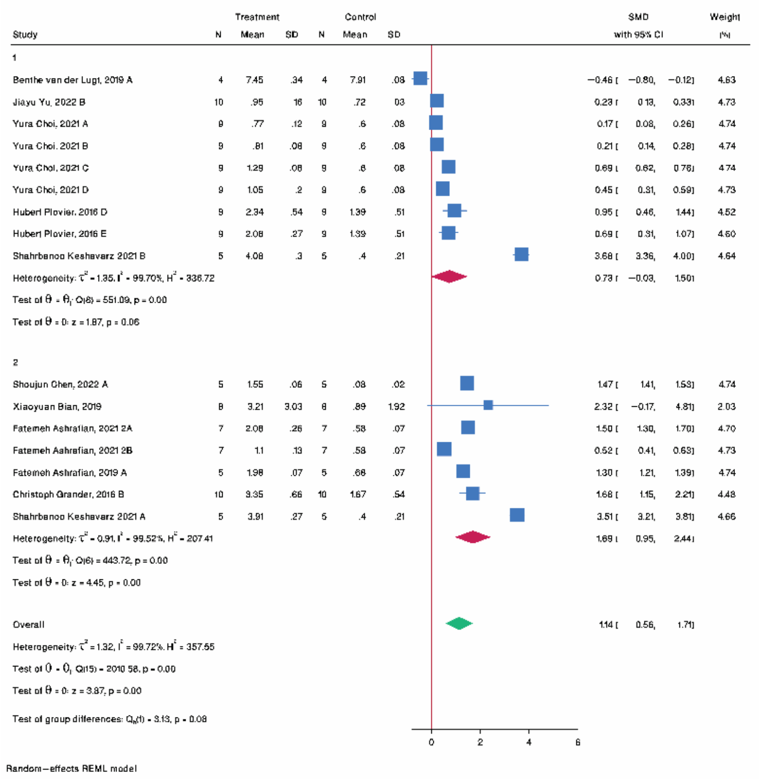

Duration

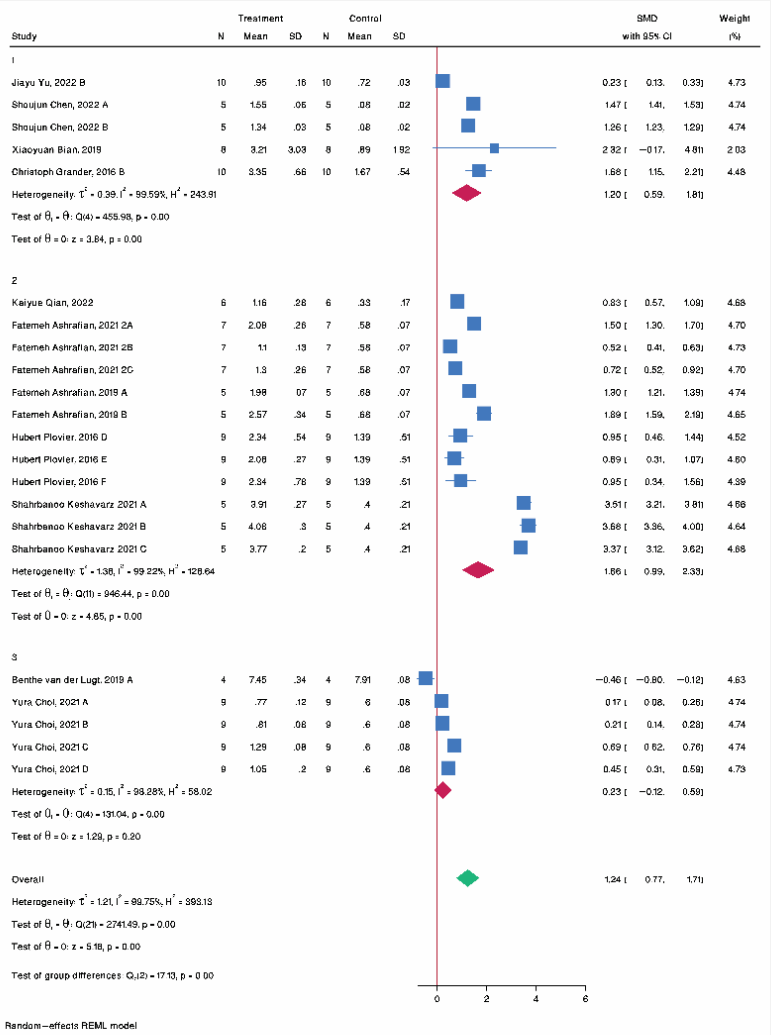

Treatment
Control
SMD with 95% CI
Weight (%)
Study N Mean SD N Mean SD
1
Jiayu Yu, 2022 B 10 .95 .18 10 .72 .03 0.23 [0.13, 0.33] 4.73
Shoujun Chen, 2022 A 5 1.55 .06 5 .08 .02 1.47 [1.41, 1.53] 4.74
Shoujun Chen, 2022 B 5 1.34 .03 5 .08 .02 1.26 [1.23, 1.29] 4.74
Xiaoyuan Bian, 2019 8 3.21 3.03 8 .89 1.92 2.32 [−0.17, 4.81] 2.03
Christoph Grander, 2016 B 10 3.35 .66 10 1.67 .54 1.68 [1.15, 2.21] 4.48
Heterogeneity: τ² = 0.39, I² = 99.59%, H² = 243.91 1.20 [0.59, 1.81]
Test of θi = θj: Q(4) = 455.98, p = 0.00
Test of θ = 0: z = 3.84, p = 0.00
2
Kaiyue Qian, 2022 6 1.16 .28 6 .33 .17 0.83 [0.57, 1.09] 4.68
Fatemeh Ashrafian, 2021 2A 7 2.08 .26 7 .58 .07 1.50 [1.30, 1.70] 4.70
Fatemeh Ashrafian, 2021 2B 7 1.1 .13 7 .58 .07 0.52 [0.41, 0.63] 4.73
Fatemeh Ashrafian, 2021 2C 7 1.3 .26 7 .58 .07 0.72 [0.52, 0.92] 4.70
Fatemeh Ashrafian, 2019 A 5 1.98 .07 5 .68 .07 1.30 [1.21, 1.39] 4.74
Fatemeh Ashrafian, 2019 B 5 2.57 .34 5 .68 .07 1.89 [1.59, 2.19] 4.65
Hubert Plovier, 2016 D 9 2.34 .54 9 1.39 .51 0.95 [0.46, 1.44] 4.52
Hubert Plovier, 2016 E 9 2.08 .27 9 1.39 .51 0.69 [0.31, 1.07] 4.60
Hubert Plovier, 2016 F 9 2.34 .78 9 1.39 .51 0.95 [0.34, 1.56] 4.39
Shahrbanoo Keshavarz 2021 A 5 3.91 .27 5 .4 .21 3.51 [3.21, 3.81] 4.66
Shahrbanoo Keshavarz 2021 B 5 4.08 .3 5 .4 .21 3.68 [3.36, 4.00] 4.64
Shahrbanoo Keshavarz 2021 C 5 3.77 .2 5 .4 .21 3.37 [3.12, 3.62] 4.68
Heterogeneity: τ² = 1.38, I² = 99.22%, H² = 128.64 1.66 [0.99, 2.33]
Test of θi = θj: Q(11) = 946.44, p = 0.00
Test of θ = 0: z = 4.85, p = 0.00
3
Benthe van der Lugt, 2019 A 4 7.45 .34 4 7.91 .08 −0.46 [−0.80, −0.12] 4.63
Yura Choi, 2021 A 9 .77 .12 9 .6 .08 0.17 [0.08, 0.26] 4.74
Yura Choi, 2021 B 9 .81 .08 9 .6 .08 0.21 [0.14, 0.28] 4.74
Yura Choi, 2021 C 9 1.29 .08 9 .6 .08 0.69 [0.62, 0.76] 4.74
Yura Choi, 2021 D 9 1.05 .2 9 .6 .08 0.45 [0.31, 0.59] 4.73
Heterogeneity: τ² = 0.15, I² = 98.28%, H² = 58.02 0.23 [−0.12, 0.59]
Test of θi = θj: Q(4) = 131.04, p = 0.00
Test of θ = 0: z = 1.29, p = 0.20
Overall 1.24 [0.77, 1.71]
Heterogeneity: τ² = 1.21, I² = 99.75%, H² = 398.13
Test of θi = θj: Q(21) = 2741.49, p = 0.00
Test of θ = 0: z = 5.18, p = 0.00
Test of group differences: Qb(2) = 17.13, p = 0.00
0
2
4
6
Random−effects REML model

Occludin

Supplement type

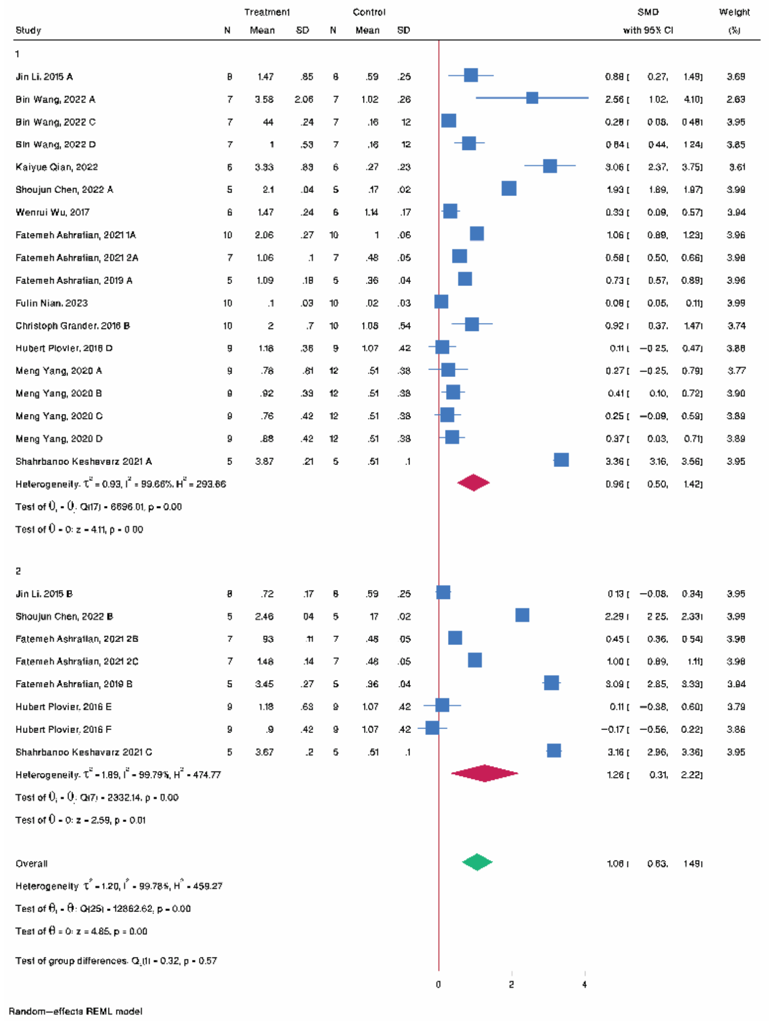

Dose

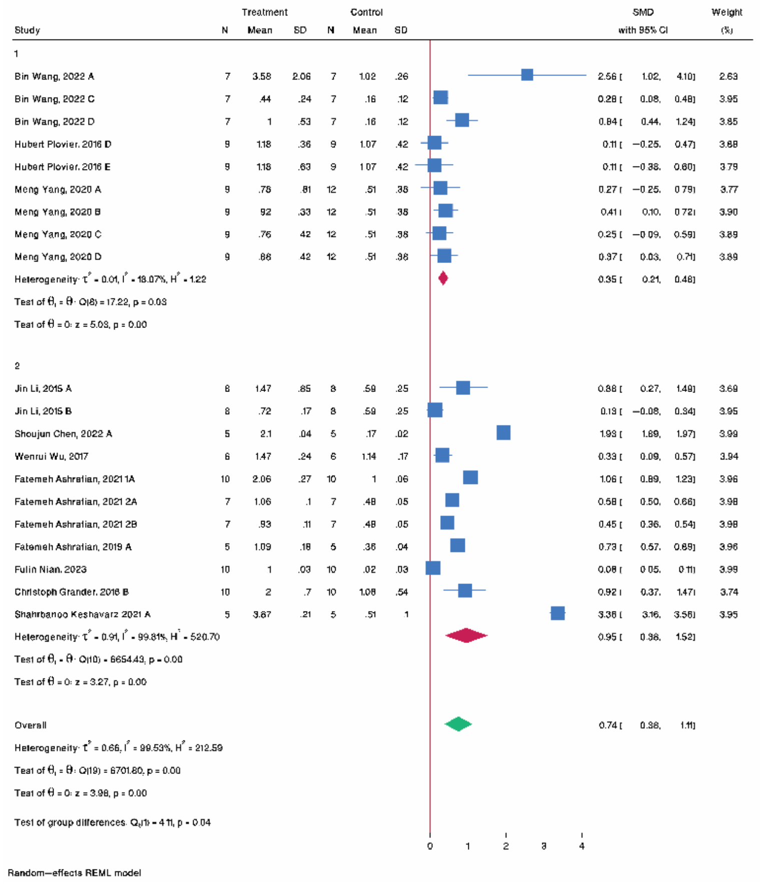

Duration

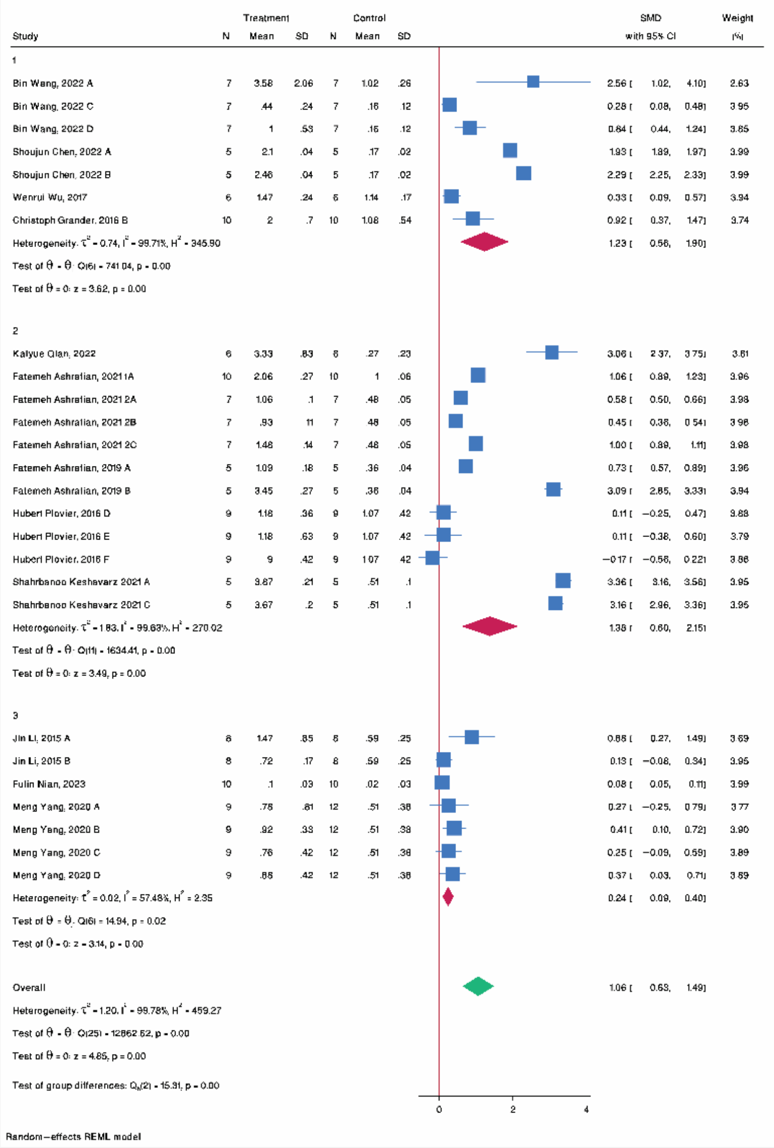

Treatment
Control
SMD with 95% CI
Weight (%)
Study
N
Mean
SD
N
Mean
SD
1
Bin Wang, 2022 A 7 3.58 2.06 7 1.02 .26 2.56 [1.02, 4.10] 2.63
Bin Wang, 2022 C 7 .44 .24 7 .16 .12 0.28 [0.08, 0.48] 3.95
Bin Wang, 2022 D 7 1 .53 7 .16 .12 0.64 [0.44, 1.24] 3.65
Shoujun Chen, 2022 A 5 2.1 .04 5 .17 .02 1.93 [1.89, 1.97] 3.99
Shoujun Chen, 2022 B 5 2.46 .04 5 .17 .02 2.29 [2.25, 2.33] 3.99
Wenrui Wu, 2017 6 1.47 .24 6 1.14 .17 0.33 [0.09, 0.57] 3.94
Christoph Grander, 2016 B 10 2 .7 10 1.08 .54 0.92 [0.37, 1.47] 3.74
Heterogeneity: τ² = 0.74, I² = 99.71%, H² = 345.90 1.23 [0.56, 1.90]
Test of θ = θ: Q(6) = 741.04, p = 0.00
Test of θ = 0: z = 3.62, p = 0.00
2
Kaiyue Qian, 2022 6 3.33 .83 6 .27 .23 3.06 [2.37, 3.75] 3.81
Fatemeh Ashrafian, 2021 1A 10 2.06 .27 10 1 .06 1.06 [0.89, 1.23] 3.96
Fatemeh Ashrafian, 2021 2A 7 1.06 .1 7 .48 .05 0.58 [0.50, 0.66] 3.98
Fatemeh Ashrafian, 2021 2B 7 .93 .11 7 .48 .05 0.45 [0.36, 0.54] 3.98
Fatemeh Ashrafian, 2021 2C 7 1.48 .14 7 .48 .05 1.00 [0.89, 1.11] 3.98
Fatemeh Ashrafian, 2019 A 5 1.09 .18 5 .36 .04 0.73 [0.57, 0.89] 3.96
Fatemeh Ashrafian, 2019 B 5 3.45 .27 5 .36 .04 3.09 [2.85, 3.33] 3.94
Hubert Plovier, 2016 D 9 1.18 .36 9 1.07 .42 0.11 [−0.25, 0.47] 3.88
Hubert Plovier, 2016 E 9 1.18 .63 9 1.07 .42 0.11 [−0.38, 0.60] 3.79
Hubert Plovier, 2016 F 9 .9 .42 9 1.07 .42 −0.17 [−0.56, 0.22] 3.86
Shahrbanoo Keshavarz 2021 A 5 3.87 .21 5 .51 .1 3.36 [3.16, 3.56] 3.95
Shahrbanoo Keshavarz 2021 C 5 3.67 .2 5 .51 .1 3.16 [2.96, 3.36] 3.95
Heterogeneity: τ² = 1.83, I² = 99.63%, H² = 270.02 1.38 [0.60, 2.15]
Test of θ = θ: Q(11) = 1634.41, p = 0.00
Test of θ = 0: z = 3.49, p = 0.00
3
Jin Li, 2015 A 8 1.47 .85 8 .59 .25 0.88 [0.27, 1.49] 3.69
Jin Li, 2015 B 8 .72 .17 8 .59 .25 0.13 [−0.08, 0.34] 3.95
Fulin Nian, 2023 10 .1 .03 10 .02 .03 0.08 [0.05, 0.11] 3.99
Meng Yang, 2020 A 9 .78 .81 12 .51 .38 0.27 [−0.25, 0.79] 3.77
Meng Yang, 2020 B 9 .92 .33 12 .51 .38 0.41 [0.10, 0.72] 3.90
Meng Yang, 2020 C 9 .76 .42 12 .51 .38 0.25 [−0.09, 0.59] 3.89
Meng Yang, 2020 D 9 .88 .42 12 .51 .38 0.37 [0.03, 0.71] 3.89
Heterogeneity: τ² = 0.02, I² = 57.48%, H² = 2.35 0.24 [0.09, 0.40]
Test of θ = θ: Q(6) = 14.94, p = 0.02
Test of θ = 0: z = 3.14, p = 0.00
Overall 1.06 [0.63, 1.49]
Heterogeneity: τ² = 1.20, I² = 99.78%, H² = 459.27
Test of θ = θ: Q(25) = 12862.52, p = 0.00
Test of θ = 0: z = 4.85, p = 0.00
Test of group differences: Q(2) = 15.91, p = 0.00
0
2
4
Random−effects REML model

Colon length

Supplement type

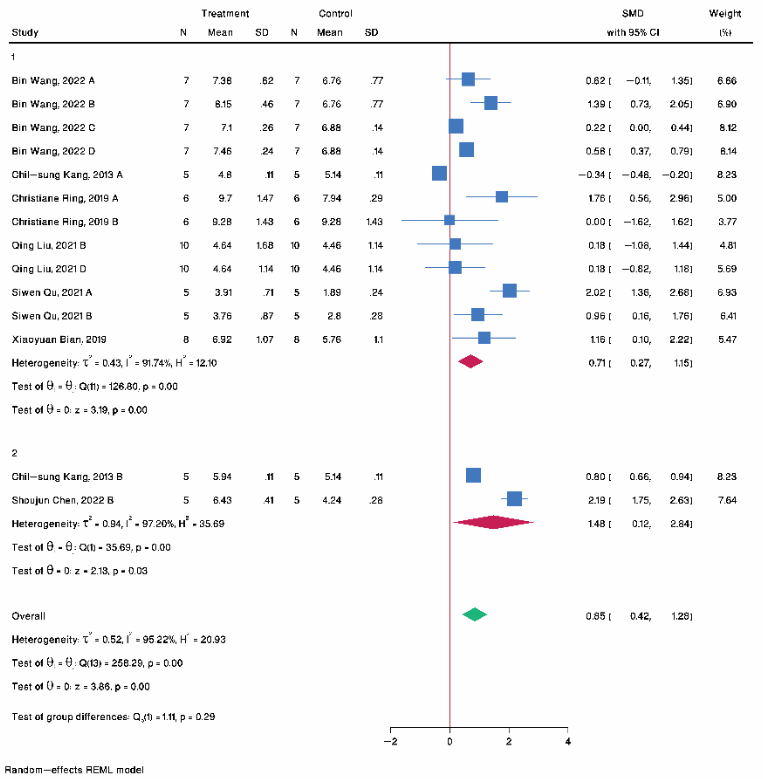

Dose

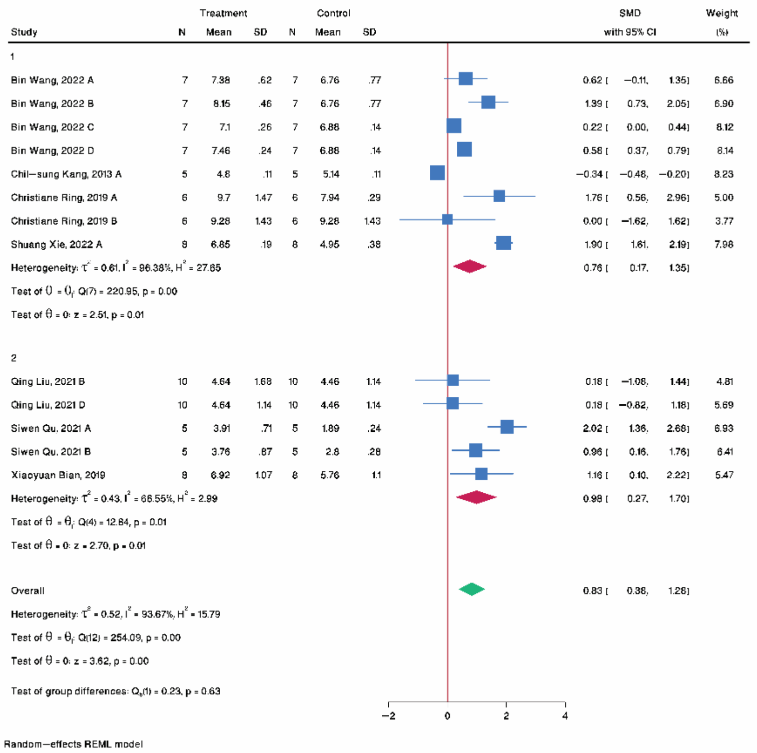

Mucus thickness

Supplement type

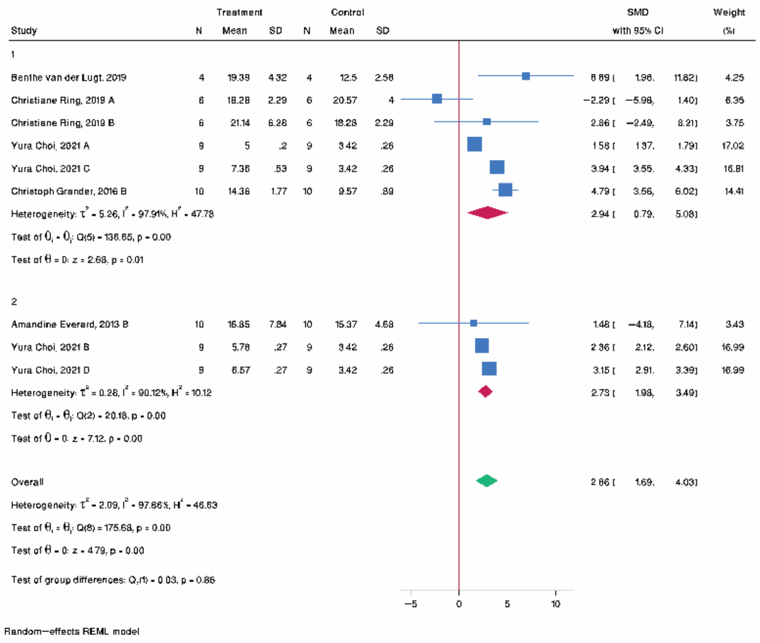

Duration

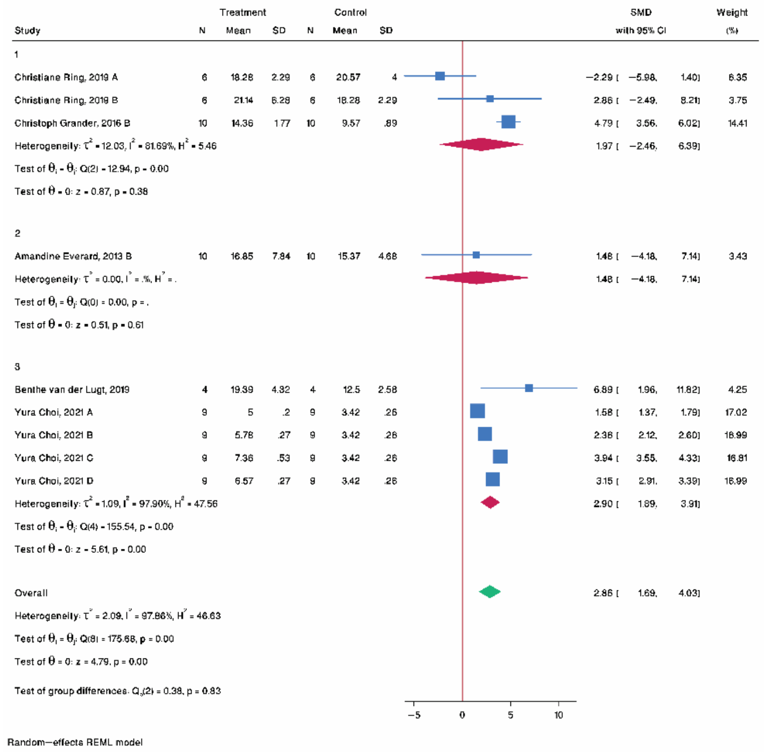

TLR-2

Supplement type

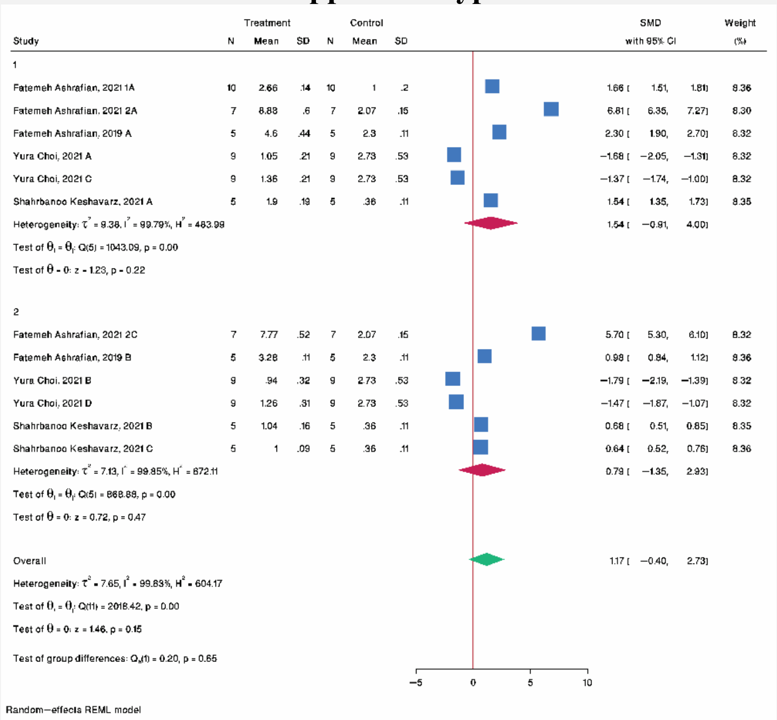

Liver enzymes

ALT

Supplement type

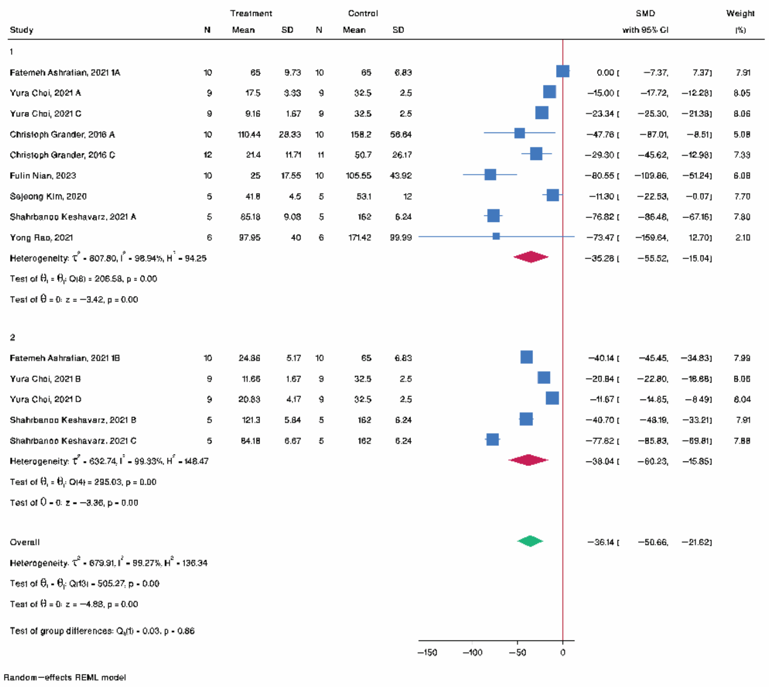

Dose

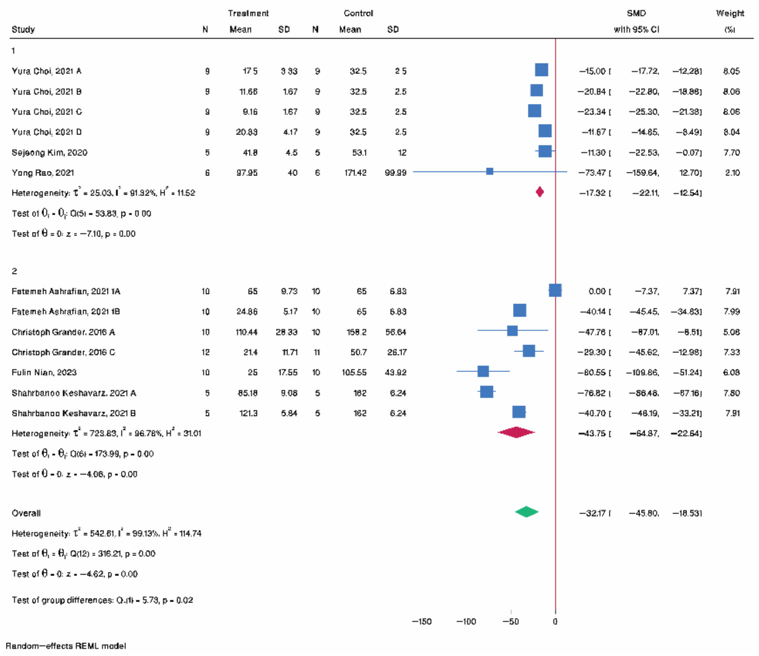

Duration

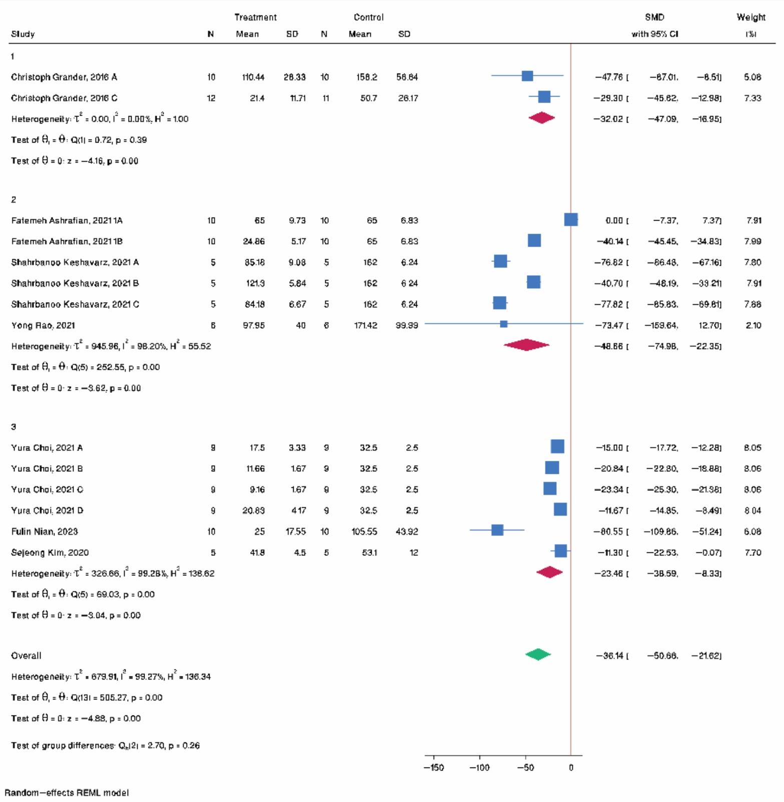

Treatment
Control
Study N Mean SD N Mean SD
SMD with 95% CI
Weight (%)
1
Christoph Grander, 2016 A 10 110.44 28.33 10 158.2 56.84 −47.76 [−87.01, −8.51] 5.06
Christoph Grander, 2016 C 12 21.4 11.71 11 50.7 26.17 −29.30 [−45.62, −12.98] 7.33
Heterogeneity: τ² = 0.00, I² = 0.00%, H² = 1.00 −32.02 [−47.09, −16.95]
Test of θi = θj: Q(1) = 0.72, p = 0.39
Test of θ = 0: z = −4.16, p = 0.00
2
Fatemeh Ashrafian, 2021 1A 10 65 9.73 10 65 6.83 0.00 [−7.37, 7.37] 7.91
Fatemeh Ashrafian, 2021 1B 10 24.86 5.17 10 65 6.83 −40.14 [−45.45, −34.83] 7.99
Shahrbanoo Keshavarz, 2021 A 5 85.18 9.08 5 162 6.24 −76.82 [−86.48, −67.16] 7.80
Shahrbanoo Keshavarz, 2021 B 5 121.3 5.84 5 162 6.24 −40.70 [−48.19, −33.21] 7.91
Shahrbanoo Keshavarz, 2021 C 5 84.18 6.67 5 162 6.24 −77.82 [−85.83, −69.81] 7.88
Yong Rao, 2021 6 97.95 40 6 171.42 99.99 −73.47 [−159.64, 12.70] 2.10
Heterogeneity: τ² = 945.96, I² = 98.20%, H² = 55.52 −48.66 [−74.98, −22.35]
Test of θi = θj: Q(5) = 252.55, p = 0.00
Test of θ = 0: z = −3.62, p = 0.00
3
Yura Choi, 2021 A 9 17.5 3.33 9 32.5 2.5 −15.00 [−17.72, −12.28] 8.05
Yura Choi, 2021 B 9 11.66 1.67 9 32.5 2.5 −20.84 [−22.80, −18.88] 8.06
Yura Choi, 2021 C 9 9.16 1.67 9 32.5 2.5 −23.34 [−25.30, −21.38] 8.06
Yura Choi, 2021 D 9 20.83 4.17 9 32.5 2.5 −11.67 [−14.85, −8.49] 8.04
Fulin Nian, 2023 10 25 17.55 10 105.55 43.92 −80.55 [−109.86, −51.24] 6.08
Sejeong Kim, 2020 5 41.8 4.5 5 53.1 12 −11.30 [−22.53, −0.07] 7.70
Heterogeneity: τ² = 326.66, I² = 99.28%, H² = 138.62 −23.46 [−38.59, −8.33]
Test of θi = θj: Q(5) = 69.03, p = 0.00
Test of θ = 0: z = −3.04, p = 0.00
Overall −36.14 [−50.66, −21.62]
Heterogeneity: τ² = 679.91, I² = 99.27%, H² = 136.34
Test of θi = θj: Q(13) = 505.27, p = 0.00
Test of θ = 0: z = −4.88, p = 0.00
Test of group differences: Qb(2) = 2.70, p = 0.26
−150 −100 −50 0
Random−effects REML model

AST

Supplement type

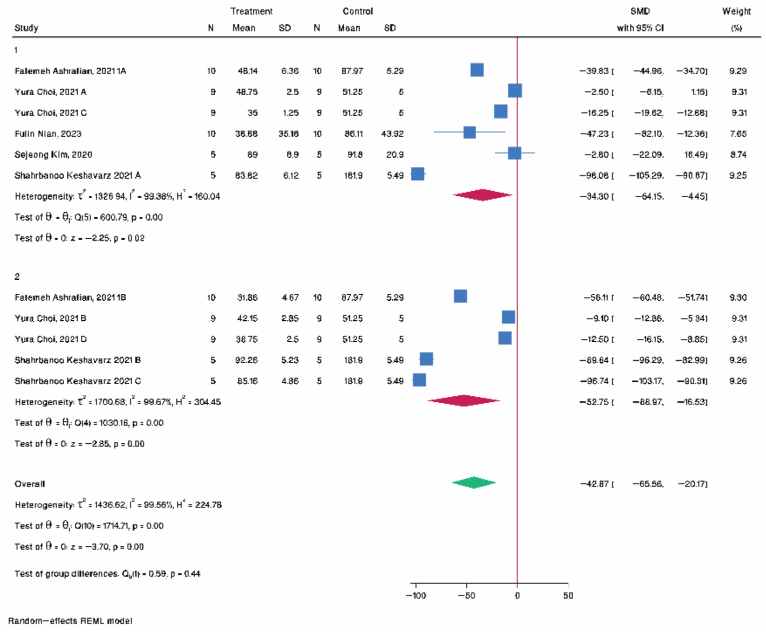

Dose

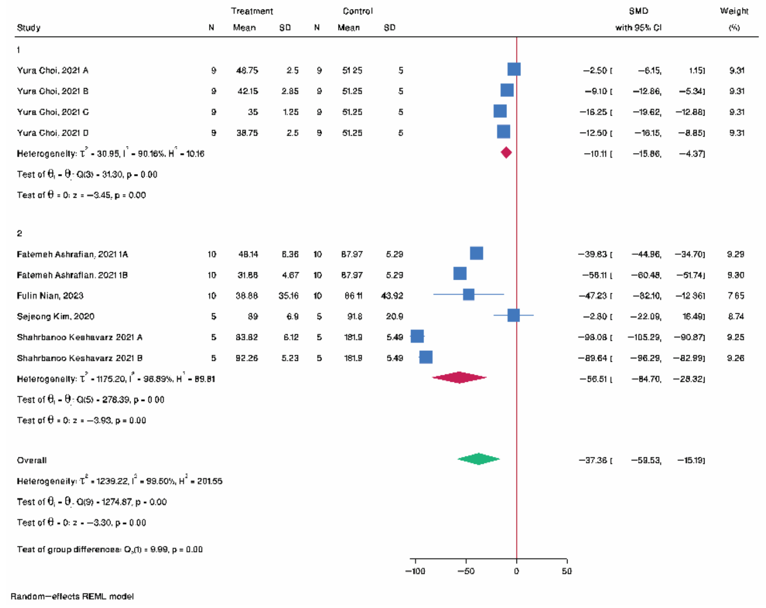

Duration

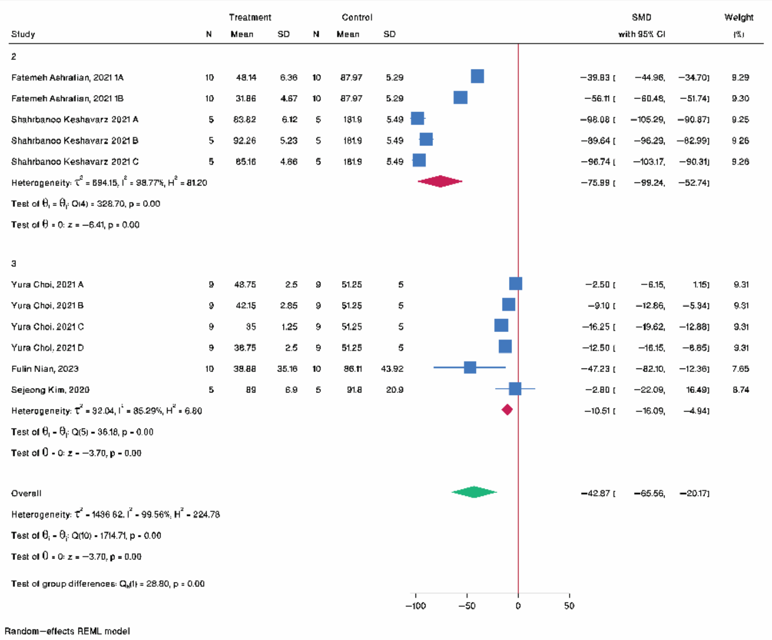

Treatment
Control
Study
N
Mean
SD
SMD
with 95% CI
Weight
(%)
2
Fatemeh Ashrafian, 2021 1A 10 48.14 6.36 10 87.97 5.29 −39.83 [−44.96, −34.70] 9.29
Fatemeh Ashrafian, 2021 1B 10 31.86 4.67 10 87.97 5.29 −56.11 [−60.48, −51.74] 9.30
Shahrbanoo Keshavarz 2021 A 5 83.82 6.12 5 181.9 5.49 −98.08 [−105.29, −90.87] 9.25
Shahrbanoo Keshavarz 2021 B 5 92.26 5.23 5 181.9 5.49 −89.64 [−96.29, −82.99] 9.26
Shahrbanoo Keshavarz 2021 C 5 85.16 4.66 5 181.9 5.49 −96.74 [−103.17, −90.31] 9.26
Heterogeneity: τ² = 694.15, I² = 98.77%, H² = 81.20 −75.99 [−99.24, −52.74]
Test of θi = θj: Q(4) = 328.70, p = 0.00
Test of θ = 0: z = −6.41, p = 0.00
3
Yura Choi, 2021 A 9 48.75 2.5 9 51.25 5 −2.50 [−6.15, 1.15] 9.31
Yura Choi, 2021 B 9 42.15 2.85 9 51.25 5 −9.10 [−12.86, −5.34] 9.31
Yura Choi, 2021 C 9 35 1.25 9 51.25 5 −16.25 [−19.62, −12.88] 9.31
Yura Choi, 2021 D 9 38.75 2.5 9 51.25 5 −12.50 [−16.15, −8.85] 9.31
Fulin Nian, 2023 10 38.88 35.16 10 86.11 43.92 −47.23 [−82.10, −12.36] 7.65
Sejeong Kim, 2020 5 89 6.9 5 91.8 20.9 −2.80 [−22.09, 16.49] 8.74
Heterogeneity: τ² = 32.04, I² = 85.29%, H² = 6.80 −10.51 [−16.09, −4.94]
Test of θi = θj: Q(5) = 36.18, p = 0.00
Test of θ = 0: z = −3.70, p = 0.00
Overall −42.87 [−65.56, −20.17]
Heterogeneity: τ² = 1436.62, I² = 99.56%, H² = 224.78
Test of θi = θj: Q(10) = 1714.71, p = 0.00
Test of θ = 0: z = −3.70, p = 0.00
Test of group differences: Qb(1) = 28.80, p = 0.00
−100
−50
0
50
Random−effects REML model

Metabolic profile

Blood glucose

Supplement type

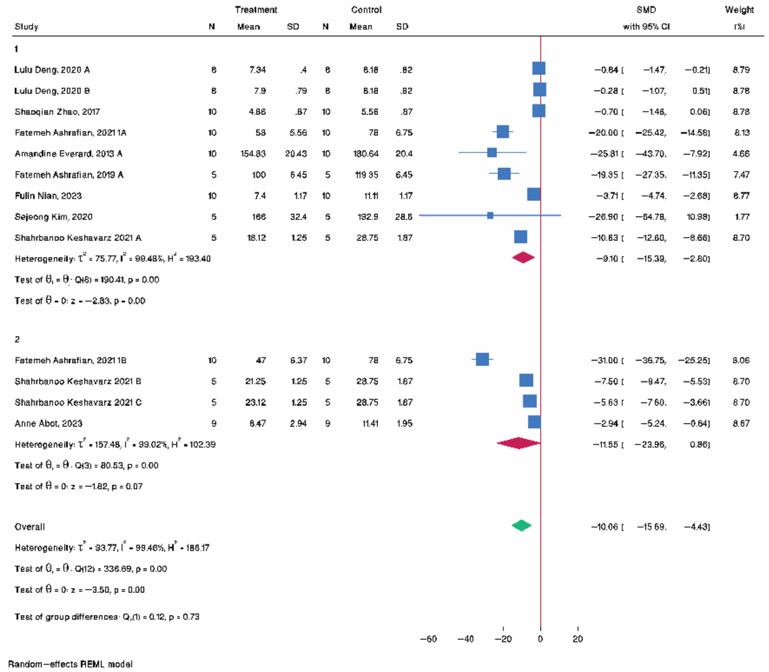

Dose

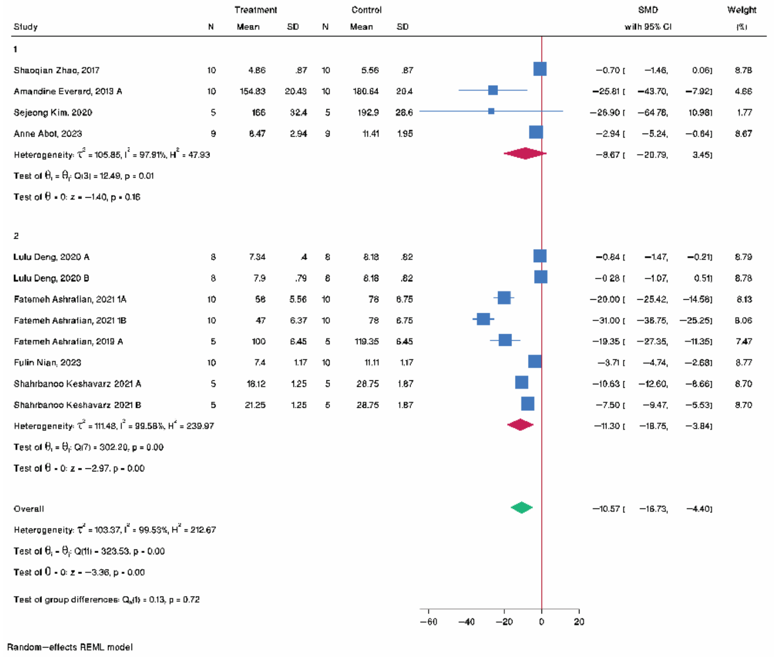

Duration

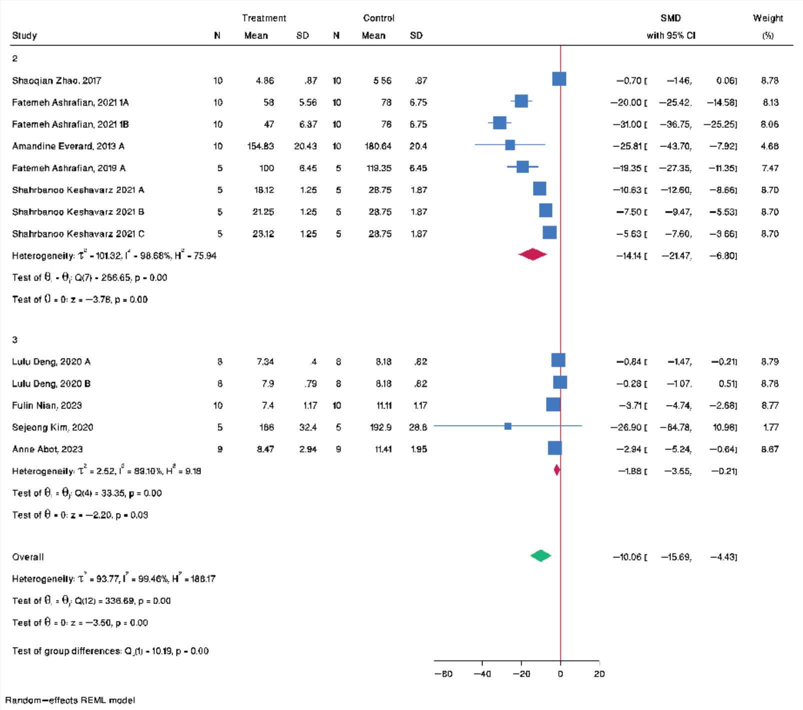
Treatment
Control
Study N Mean SD N Mean SD
SMD with 95% CI
Weight (%)
2
Shaoqian Zhao, 2017 10 4.86 .87 10 5 56 .87 −0.70 [−1.46, 0.06] 8.78
Fatemeh Ashrafian, 2021 1A 10 58 5.56 10 78 6.75 −20.00 [−25.42, −14.58] 8.13
Fatemeh Ashrafian, 2021 1B 10 47 6.37 10 76 6.75 −31.00 [−36.75, −25.25] 8.06
Amandine Everard, 2013 A 10 154.83 20.43 10 180.64 20.4 −25.81 [−43.70, −7.92] 4.68
Fatemeh Ashrafian, 2019 A 5 100 6.45 5 119.35 6.45 −19.35 [−27.35, −11.35] 7.47
Shahrbanoo Keshavarz 2021 A 5 18.12 1.25 5 28.75 1.87 −10.63 [−12.60, −8.66] 8.70
Shahrbanoo Keshavarz 2021 B 5 21.25 1.25 5 28.75 1.87 −7.50 [−9.47, −5.53] 8.70
Shahrbanoo Keshavarz 2021 C 5 23.12 1.25 5 28.75 1.87 −5.63 [−7.60, −3.66] 8.70
Heterogeneity: τ² = 101.32, I² = 98.68%, H² = 75.94 −14.14 [−21.47, −6.80]
Test of θi = θj: Q(7) = 266.65, p = 0.00
Test of θ = 0: z = −3.78, p = 0.00
3
Lulu Deng, 2020 A 8 7.34 .4 8 8.18 .82 −0.84 [−1.47, −0.21] 8.79
Lulu Deng, 2020 B 8 7.9 .79 8 8.18 .82 −0.28 [−1.07, 0.51] 8.78
Fulin Nian, 2023 10 7.4 1.17 10 11.11 1.17 −3.71 [−4.74, −2.68] 8.77
Sejeong Kim, 2020 5 166 32.4 5 192.9 28.8 −26.90 [−64.78, 10.96] 1.77
Anne Abot, 2023 9 8.47 2.94 9 11.41 1.95 −2.94 [−5.24, −0.64] 8.67
Heterogeneity: τ² = 2.52, I² = 89.10%, H² = 9.18 −1.88 [−3.55, −0.21]
Test of θi = θj: Q(4) = 33.35, p = 0.00
Test of θ = 0: z = −2.20, p = 0.03
Overall −10.06 [−15.69, −4.43]
Heterogeneity: τ² = 93.77, I² = 99.46%, H² = 186.17
Test of θi = θj: Q(12) = 336.69, p = 0.00
Test of θ = 0: z = −3.50, p = 0.00
Test of group differences: Qb(1) = 10.19, p = 0.00
−60 −40 −20 0 20
Random−effects REML model

Total cholesterol

Supplement type

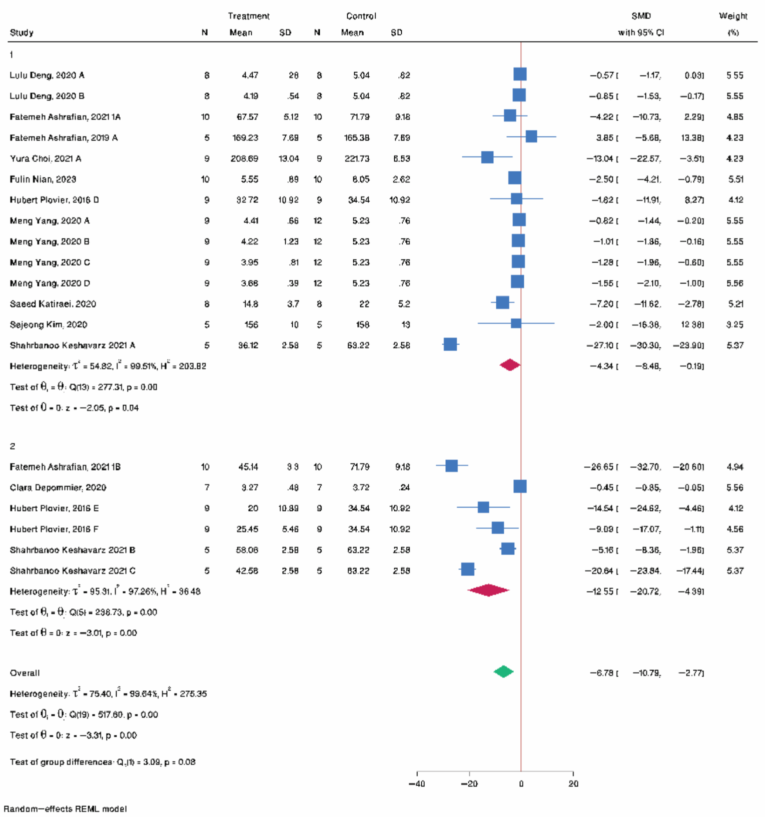

Dose

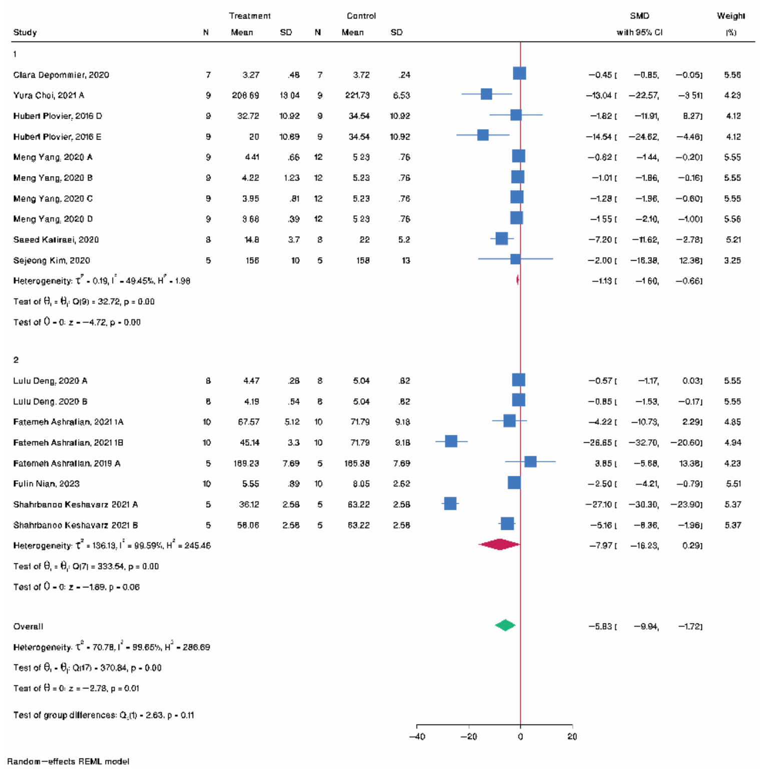

Duration

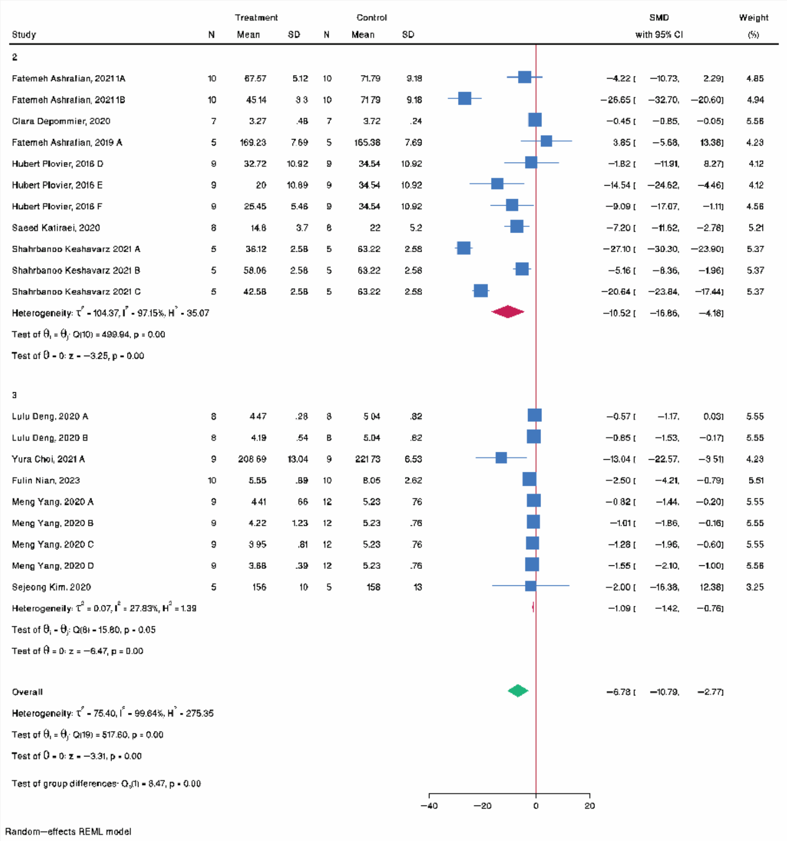
Treatment
Control
Study
N
Mean
SD
N
Mean
SD
SMD with 95% CI
Weight (%)
2
Fatemeh Ashrafian, 2021 1A 10 67.57 5.12 10 71.79 9.18 −4.22 [−10.73, 2.29] 4.85
Fatemeh Ashrafian, 2021 1B 10 45 14 3 3 10 71 79 9.18 −26.65 [−32.70, −20.60] 4.94
Clara Depommier, 2020 7 3.27 .48 7 3.72 .24 −0.45 [−0.85, −0.05] 5.58
Fatemeh Ashrafian, 2019 A 5 169.23 7.69 5 165.38 7.69 3.85 [−5.68, 13.38] 4.23
Hubert Plovier, 2016 D 9 32.72 10.92 9 34.54 10.92 −1.82 [−11.91, 8.27] 4.12
Hubert Plovier, 2016 E 9 20 10.69 9 34.54 10.92 −14.54 [−24.62, −4.46] 4.12
Hubert Plovier, 2016 F 9 25.45 5.46 9 34.54 10.92 −9.09 [−17.07, −1.11] 4.56
Saeed Katiraei, 2020 8 14.6 3.7 8 22 5.2 −7.20 [−11.62, −2.78] 5.21
Shahrbanoo Keshavarz 2021 A 5 36.12 2.58 5 63.22 2.58 −27.10 [−30.30, −23.90] 5.37
Shahrbanoo Keshavarz 2021 B 5 58.06 2.58 5 63.22 2.58 −5.16 [−8.36, −1.96] 5.37
Shahrbanoo Keshavarz 2021 C 5 42.58 2.58 5 63.22 2.58 −20.64 [−23.84, −17.44] 5.37
Heterogeneity: τ² = 104.37, I² = 97.15%, H² = 35.07 −10.52 [−16.86, −4.18]
Test of θi = θj: Q(10) = 499.94, p = 0.00
Test of θ = 0: z = −3.25, p = 0.00
3
Lulu Deng, 2020 A 8 4 47 .28 8 5 04 .82 −0.57 [−1.17, 0.03] 5.55
Lulu Deng, 2020 B 8 4.19 .54 8 5.04 .82 −0.85 [−1.53, −0.17] 5.55
Yura Choi, 2021 A 9 208 69 13.04 9 221 73 6.53 −13.04 [−22.57, −3 51] 4.23
Fulin Nian, 2023 10 5.55 .89 10 8.05 2.62 −2.50 [−4.21, −0.79] 5.51
Meng Yang, 2020 A 9 4 41 66 12 5.23 76 −0 82 [−1.44, −0.20] 5.55
Meng Yang, 2020 B 9 4.22 1.23 12 5.23 .76 −1.01 [−1.86, −0.16] 5.55
Meng Yang, 2020 C 9 3 95 .81 12 5.23 .76 −1.28 [−1.96, −0.60] 5.55
Meng Yang, 2020 D 9 3.68 .39 12 5.23 .76 −1.55 [−2.10, −1.00] 5.58
Sejeong Kim, 2020 5 156 10 5 158 13 −2.00 [−16.38, 12.38] 3.25
Heterogeneity: τ² = 0.07, I² = 27.83%, H² = 1.39 −1.09 [−1.42, −0.76]
Test of θi = θj: Q(8) = 15.60, p = 0.05
Test of θ = 0: z = −6.47, p = 0.00
Overall −6.78 [−10.79, −2.77]
Heterogeneity: τ² = 75.40, I² = 99.64%, H² = 275.35
Test of θi = θj: Q(19) = 517.60, p = 0.00
Test of θ = 0: z = −3.31, p = 0.00
Test of group differences: Qb(1) = 8.47, p = 0.00
−40
−20
0
20
Random−effects REML model

TG

Supplement type

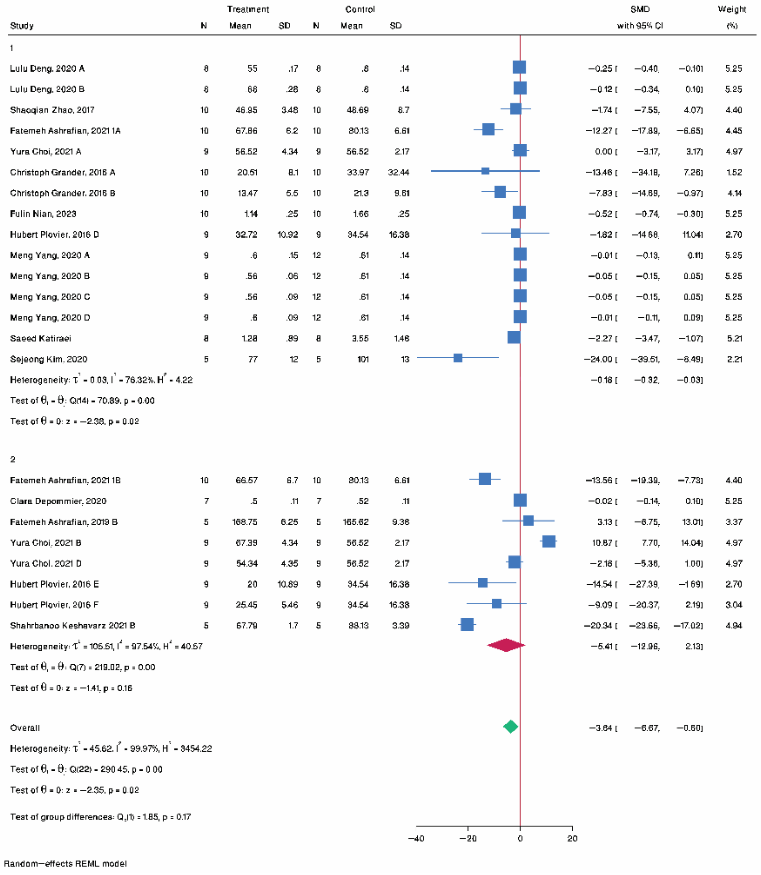

Dose

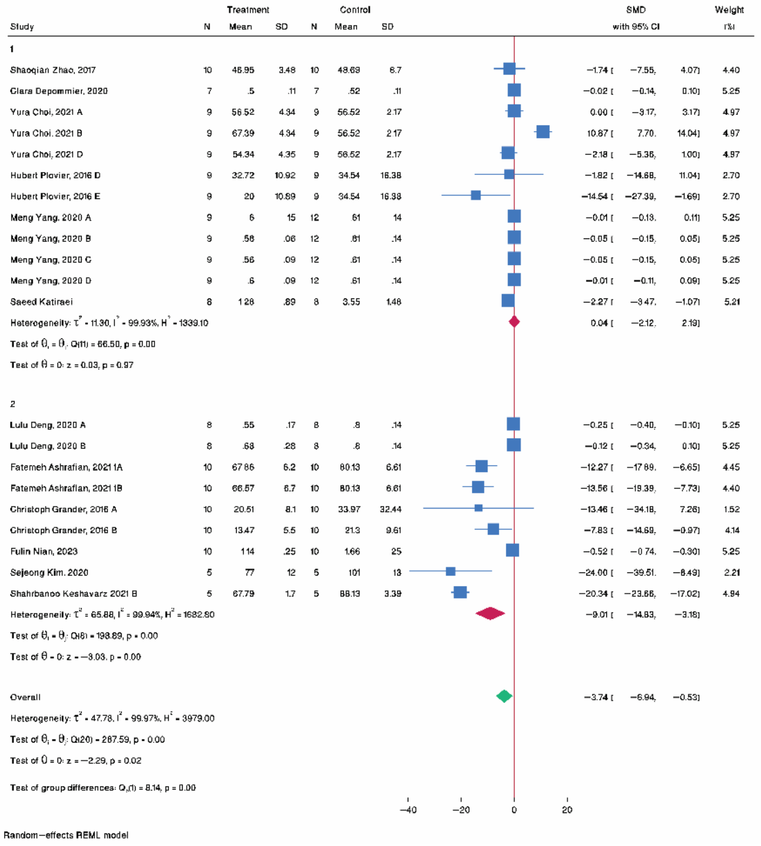

Duration

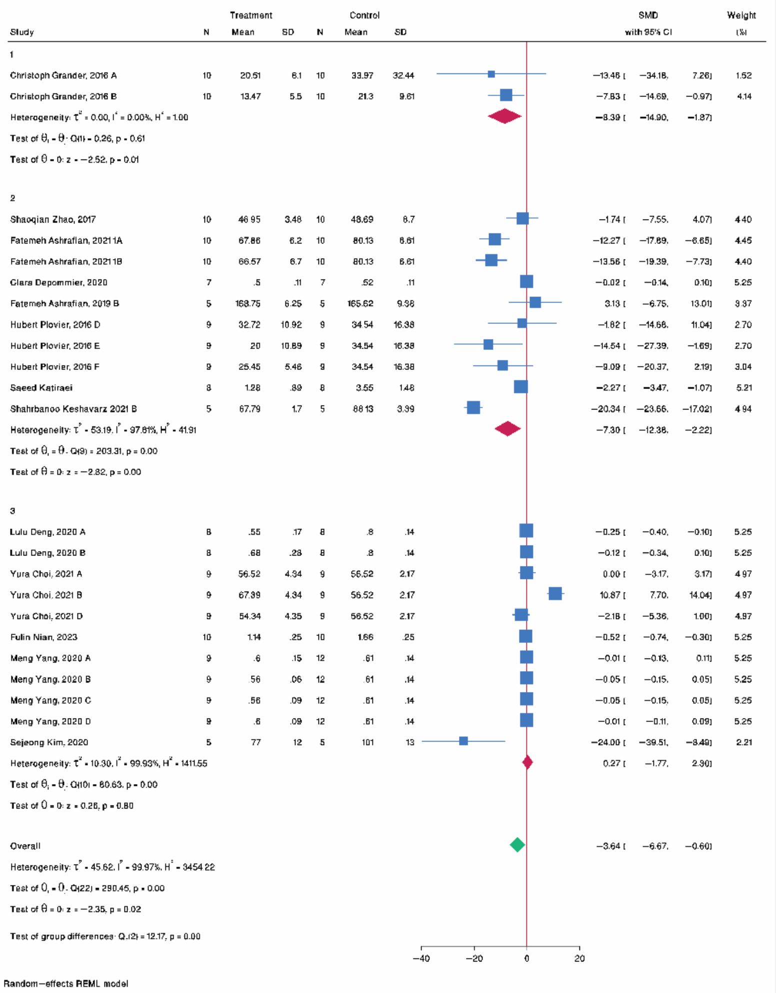
Treatment
Control
SMD with 95% CI
Weight (%)
Study N Mean SD N Mean SD
1
Christoph Grander, 2016 A 10 20.51 6.1 10 33.97 32.44 −13.46 [−34.18, 7.26] 1.52
Christoph Grander, 2016 B 10 13.47 5.5 10 21.3 9.61 −7.83 [−14.69, −0.97] 4.14
Heterogeneity: τ² = 0.00, I² = 0.00%, H² = 1.00 −8.39 [−14.90, −1.87]
Test of θi = θj: Q(1) = 0.26, p = 0.61
Test of θ = 0: z = −2.52, p = 0.01
2
Shaoqian Zhao, 2017 10 46.95 3.48 10 48.69 6.7 −1.74 [−7.55, 4.07] 4.40
Fatemeh Ashrafian, 2021 1A 10 67.86 6.2 10 80.13 6.61 −12.27 [−17.89, −6.65] 4.45
Fatemeh Ashrafian, 2021 1B 10 66.57 6.7 10 80.13 6.61 −13.56 [−19.39, −7.73] 4.40
Clara Depommier, 2020 7 .5 .11 7 .52 .11 −0.02 [−0.14, 0.10] 5.25
Fatemeh Ashrafian, 2019 B 5 168.75 6.25 5 165.62 9.38 3.13 [−6.75, 13.01] 3.37
Hubert Plovier, 2016 D 9 32.72 10.92 9 34.54 16.38 −1.82 [−14.66, 11.04] 2.70
Hubert Plovier, 2016 E 9 20 10.89 9 34.54 16.38 −14.54 [−27.39, −1.69] 2.70
Hubert Plovier, 2016 F 9 25.45 5.46 9 34.54 16.38 −9.09 [−20.37, 2.19] 3.04
Saeed Katiraei 8 1.28 .89 8 3.55 1.48 −2.27 [−3.47, −1.07] 5.21
Shahrbanoo Keshavarz 2021 B 5 67.79 1.7 5 88.13 3.39 −20.34 [−23.66, −17.02] 4.94
Heterogeneity: τ² = 53.19, I² = 97.61%, H² = 41.91 −7.30 [−12.38, −2.22]
Test of θi = θj: Q(9) = 203.31, p = 0.00
Test of θ = 0: z = −2.82, p = 0.00
3
Lulu Deng, 2020 A 8 .55 .17 8 .8 .14 −0.25 [−0.40, −0.10] 5.25
Lulu Deng, 2020 B 8 .68 .28 8 .8 .14 −0.12 [−0.34, 0.10] 5.25
Yura Choi, 2021 A 9 56.52 4.34 9 56.52 2.17 0.00 [−3.17, 3.17] 4.97
Yura Choi, 2021 B 9 67.39 4.34 9 56.52 2.17 10.87 [7.70, 14.04] 4.97
Yura Choi, 2021 D 9 54.34 4.35 9 56.52 2.17 −2.18 [−5.36, 1.00] 4.97
Fulin Nian, 2023 10 1.14 .25 10 1.66 .25 −0.52 [−0.74, −0.30] 5.25
Meng Yang, 2020 A 9 .6 .15 12 .61 .14 −0.01 [−0.13, 0.11] 5.25
Meng Yang, 2020 B 9 .56 .06 12 .61 .14 −0.05 [−0.15, 0.05] 5.25
Meng Yang, 2020 C 9 .56 .09 12 .61 .14 −0.05 [−0.15, 0.05] 5.25
Meng Yang, 2020 D 9 .6 .09 12 .61 .14 −0.01 [−0.11, 0.09] 5.25
Sejeong Kim, 2020 5 77 12 5 101 13 −24.00 [−39.51, −8.49] 2.21
Heterogeneity: τ² = 10.30, I² = 99.93%, H² = 1411.55 0.27 [−1.77, 2.30]
Test of θi = θj: Q(10) = 60.63, p = 0.00
Test of θ = 0: z = 0.26, p = 0.80
Overall −3.64 [−6.67, −0.60]
Heterogeneity: τ² = 45.62, I² = 99.97%, H² = 3454.22
Test of θi = θj: Q(22) = 290.45, p = 0.00
Test of θ = 0: z = −2.35, p = 0.02
Test of group differences: Qb(2) = 12.17, p = 0.00
−40 −20 0 20
Random−effects REML model

Body weight (GI disorders)

Dose

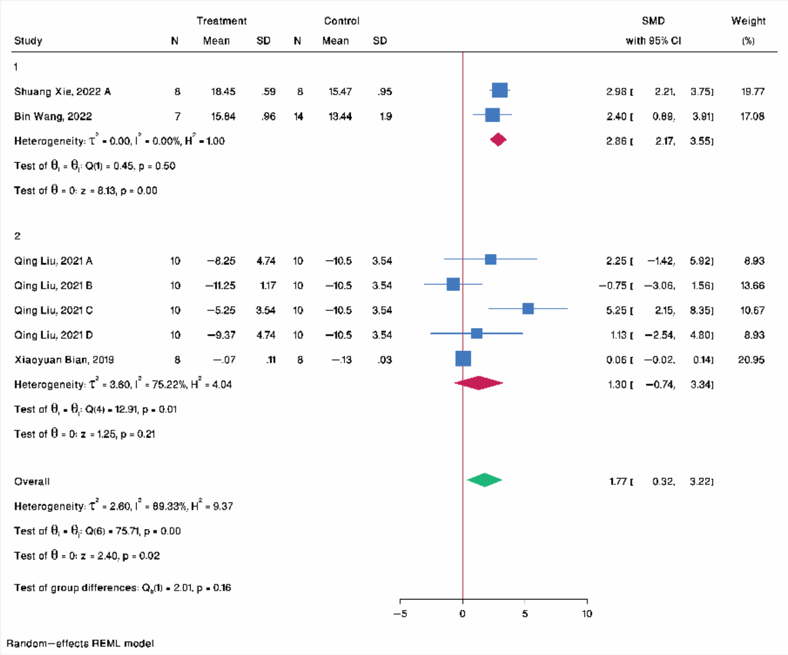

Body weight (metabolic disorders)

Supplement type

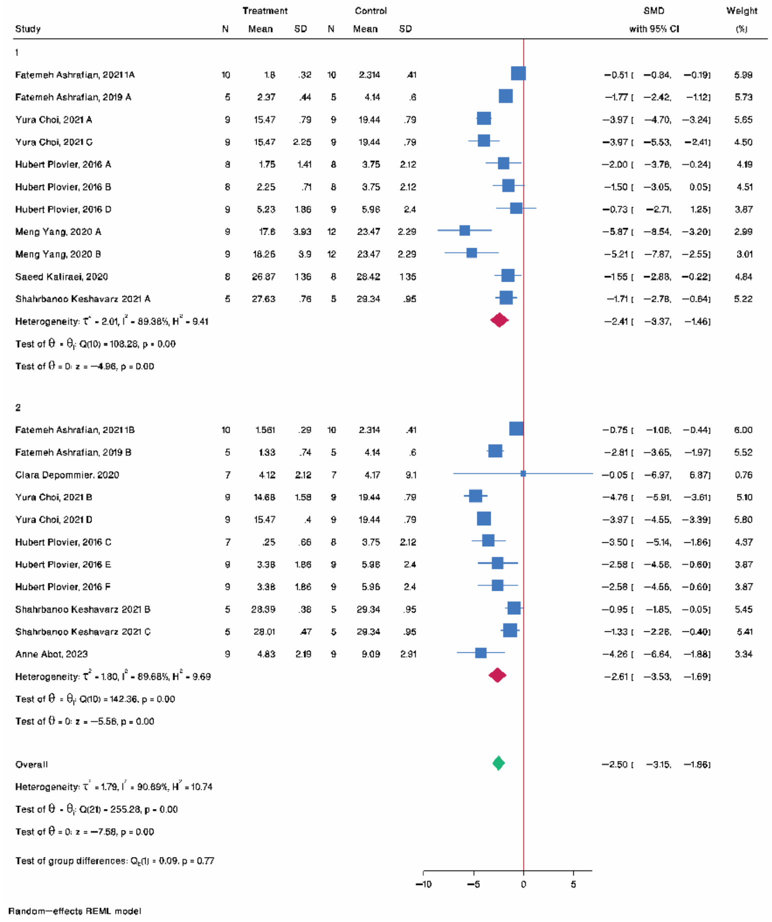

Dose

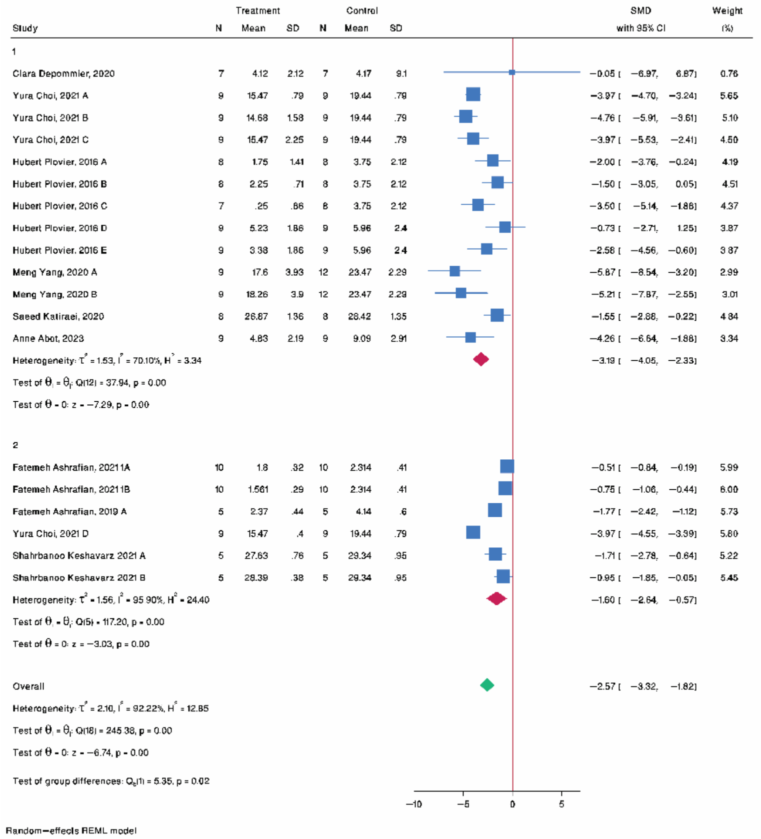

Duration

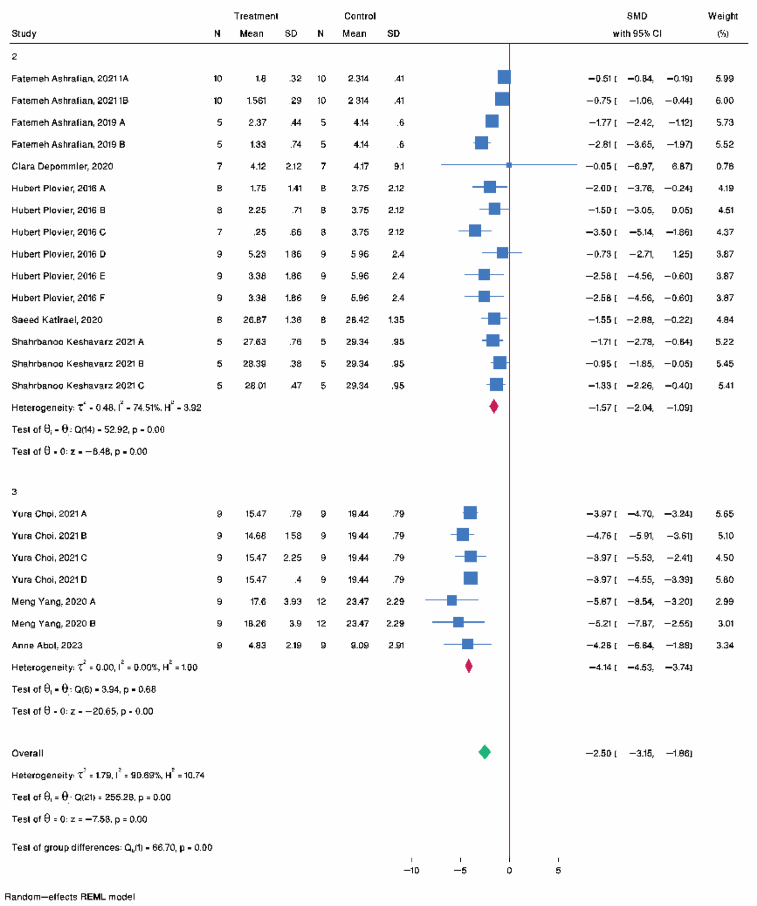

Treatment
Control
Study N Mean SD N Mean SD
SMD with 95% CI
Weight (%)
2
Fatemeh Ashrafian, 2021 1A 10 1.8 .32 10 2.314 .41 −0.51 [−0.84, −0.19] 5.99
Fatemeh Ashrafian, 2021 1B 10 1.561 .29 10 2.314 .41 −0.75 [−1.06, −0.44] 6.00
Fatemeh Ashrafian, 2019 A 5 2.37 .44 5 4.14 .6 −1.77 [−2.42, −1.12] 5.73
Fatemeh Ashrafian, 2019 B 5 1.33 .74 5 4.14 .6 −2.81 [−3.65, −1.97] 5.52
Clara Depommier, 2020 7 4.12 2.12 7 4.17 9.1 −0.05 [−6.97, 6.87] 0.76
Hubert Plovier, 2016 A 8 1.75 1.41 8 3.75 2.12 −2.00 [−3.76, −0.24] 4.19
Hubert Plovier, 2016 B 8 2.25 .71 8 3.75 2.12 −1.50 [−3.05, 0.05] 4.51
Hubert Plovier, 2016 C 7 .25 .66 8 3.75 2.12 −3.50 [−5.14, −1.86] 4.37
Hubert Plovier, 2016 D 9 5.23 1.86 9 5.96 2.4 −0.73 [−2.71, 1.25] 3.87
Hubert Plovier, 2016 E 9 3.38 1.86 9 5.96 2.4 −2.58 [−4.56, −0.60] 3.87
Hubert Plovier, 2016 F 9 3.38 1.86 9 5.96 2.4 −2.58 [−4.56, −0.60] 3.87
Saeed Katiraei, 2020 8 26.87 1.36 8 28.42 1.35 −1.55 [−2.88, −0.22] 4.84
Shahrbanoo Keshavarz 2021 A 5 27.63 .76 5 29.34 .95 −1.71 [−2.78, −0.64] 5.22
Shahrbanoo Keshavarz 2021 B 5 28.39 .38 5 29.34 .95 −0.95 [−1.85, −0.05] 5.45
Shahrbanoo Keshavarz 2021 C 5 28.01 .47 5 29.34 .95 −1.33 [−2.26, −0.40] 5.41
Heterogeneity: τ^2 = 0.48, I^2 = 74.51%, H^2 = 3.92 −1.57 [−2.04, −1.09]
Test of $\theta_i = \theta_j$: Q(14) = 52.92, p = 0.00
Test of θ = 0: z = −6.48, p = 0.00
3
Yura Choi, 2021 A 9 15.47 .79 9 19.44 .79 −3.97 [−4.70, −3.24] 5.65
Yura Choi, 2021 B 9 14.68 1.58 9 19.44 .79 −4.76 [−5.91, −3.61] 5.10
Yura Choi, 2021 C 9 15.47 2.25 9 19.44 .79 −3.97 [−5.53, −2.41] 4.50
Yura Choi, 2021 D 9 15.47 .4 9 19.44 .79 −3.97 [−4.55, −3.39] 5.80
Meng Yang, 2020 A 9 17.6 3.93 12 23.47 2.29 −5.87 [−8.54, −3.20] 2.99
Meng Yang, 2020 B 9 18.26 3.9 12 23.47 2.29 −5.21 [−7.87, −2.55] 3.01
Anne Abot, 2023 9 4.83 2.19 9 9.09 2.91 −4.26 [−6.64, −1.88] 3.34
Heterogeneity: τ^2 = 0.00, I^2 = 0.00%, H^2 = 1.00 −4.14 [−4.53, −3.74]
Test of $\theta_i = \theta_j$: Q(6) = 3.94, p = 0.68
Test of θ = 0: z = −20.65, p = 0.00
Overall −2.50 [−3.15, −1.86]
Heterogeneity: τ^2 = 1.79, I^2 = 90.69%, H^2 = 10.74
Test of $\theta_i = \theta_j$: Q(21) = 255.28, p = 0.00
Test of θ = 0: z = −7.58, p = 0.00
Test of group differences: $Q_b(1)$ = 66.70, p = 0.00
−10
−5
0
5
Random−effects REML model
